# Amylose content in rice: integrating genetics, grain filling physiology, and breeding strategies

**DOI:** 10.3389/fpls.2026.1891006

**Published:** 2026-08-07

**Authors:** Bakdaulet Usenbekov, Aliya Meldebekova, Innabat Sartbayeva, Aigul Amirova, Saule Atabayeva, Nurlan Usenbekov, Dana Mynbayeva, Taira Kurbangaliyeva

**Affiliations:** 1 Laboratory of Plant Physiology and Biochemistry, Institute of Plant Biology and Biotechnology (IPBB), Almaty, Kazakhstan; 2 Department of Molecular Biology and Genetics, Faculty of Biology and Biotechnology, Al-Farabi Kazakh National University, Almaty, Kazakhstan; 3 Department of Biotechnology, Faculty of Biology and Biotechnology, Al-Farabi Kazakh National University, Almaty, Kazakhstan

**Keywords:** amylose content, CRISPR, genome, glycemic index, marker-assisted selection, resistant starch, rice starch, Waxy gene (Wx)

## Abstract

Rice is a major staple crop worldwide, and variation in starch composition is a key determinant of grain quality, end-use functionality, and breeding value. Among starch components, amylose content (AC) plays a central role in defining the physicochemical properties of rice grains and reflects the coordinated regulation of starch biosynthesis during endosperm development. This review provides an integrated analysis of amylose variation in rice from a genetics–physiology–breeding perspective, based on a structured literature search using PubMed, Scopus, Web of Science, and Google Scholar covering studies published between 2000 and 2026. The analysis synthesizes current knowledge on the molecular regulation of amylose biosynthesis, with emphasis on the Waxy (*Wx*) gene and associated enzymes within the starch biosynthetic network, as well as on grain filling physiology, source–sink carbon partitioning, and environmental modulation of amylose accumulation. In addition, we examine natural genetic variation across rice germplasm and evaluate breeding strategies, including marker-assisted selection, genomic selection, and genome editing approaches, for optimizing amylose content and grain quality. Evidence indicates that amylose accumulation is governed by complex interactions among genetic, physiological, and environmental factors that collectively determine starch structure, grain functionality, and stability across production environments. By linking molecular mechanisms, physiological processes, and breeding strategies, this review provides a framework for the development of rice cultivars with stable amylose profiles, predictable grain quality, and improved adaptation to diverse agroecological conditions.

## 1 Introduction

Rice is one of the most important staple crops worldwide and serves as a major source of dietary energy for nearly half of the global population. The vast majority of cultivated rice belongs to *Oryza sativa* L., which dominates global production and breeding programs aimed at improving yield, resilience, and grain quality (Duan et al., 2024; Tang et al., 2024). Within *O. sativa*, the two major subspecies, *indica* and *japonica*, represent genetically differentiated groups that vary in adaptation, agronomic performance, and grain quality traits. These differences are closely associated with variation in the allelic architecture of starch biosynthesis pathways that determine eating and cooking quality (Cuevas and Fitzgerald, 2012; Li et al., 2016; Tao et al., 2019).

Starch constitutes approximately 90% of milled rice grain dry weight and is synthesized and deposited in endosperm amyloplasts during grain filling (Cuevas and Fitzgerald, 2012). It consists primarily of two glucose polymers, amylose and amylopectin, whose relative proportions and structural organization define the physicochemical properties of cooked rice, including texture, gelatinization behavior, and pasting characteristics (Butardo et al., 2019; Sharma and Khanna, 2019; Chen et al., 2022; Li et al., 2024). Among these components, amylose content (AC) is widely recognized as a key determinant of grain quality and a central target in rice breeding programs. However, AC represents only one dimension of starch functionality, which is governed by an integrated network of enzymatic processes controlling glucan elongation, branching, and granule organization.

The molecular basis of amylose biosynthesis is primarily controlled by the *Waxy* (*Wx*) gene, which encodes granule-bound starch synthase I (GBSSI), the enzyme responsible for elongating the linear glucan chains in developing endosperm (Sharma and Khanna, 2019). Natural allelic variation at the *Wx* locus, together with modifier genes involved in amylopectin biosynthesis such as starch synthases and branching enzymes, generates a continuous spectrum of starch structures and functional properties across rice germplasm (Li et al., 2016; Sharma and Khanna, 2019; Farooq and Yu, 2025). These genetic effects are further modulated by environmental factors, particularly temperature during grain filling, which influence gene expression, enzyme activity, and carbon partitioning within the endosperm (Frei et al., 2003; Fitzgerald et al., 2011; Ohtsubo, 2016; Atkinson et al., 2021). Thus, amylose accumulation is closely linked to grain filling physiology and source–sink carbon partitioning processes that determine starch deposition and final grain quality (Fasahat et al., 2014; Li et al., 2017; Li et al., 2023; Nakamura et al., 2023).

Variation in starch composition also affects digestibility and post-harvest functionality (Fu et al., 2022), although these properties depend on starch structure and processing conditions rather than amylose content alone (Wang et al., 2014; Cheng et al., 2022; Fu et al., 2022). This highlights that starch functionality reflects integrated structural and physiological determinants rather than a single compositional parameter. Despite substantial advances in rice genomics and starch biochemistry, knowledge of amylose regulation, grain filling physiology, and breeding applications has often been developed in parallel rather than within a unified framework. This fragmentation limits the translation of molecular insights into breeding strategies capable of producing rice varieties with stable grain quality across environments. In particular, strong genotype × environment interactions, especially temperature effects during grain filling, complicate the prediction of amylose content and associated functional traits.

The aim of this review is to provide an integrated analysis of amylose content in rice from a genetics–physiology–breeding perspective. We first examine the molecular regulation of amylose biosynthesis, including the role of the *Wx* gene and modifier enzymes within the starch biosynthetic network. We then discuss the structural and physicochemical basis of starch functionality and its relationship with grain quality traits. In addition, we analyze the physiological processes underlying amylose accumulation during grain filling, with emphasis on source–sink dynamics and environmental regulation. Finally, we summarize genetic resources and breeding strategies, including marker-assisted selection (MAS) and genome editing, for optimizing amylose content across diverse production systems. By linking molecular mechanisms, physiological processes, and breeding approaches, this review aims to support the development of rice varieties with predictable grain quality and stable performance under variable environmental conditions.

## 2 Methods

**Figure 1:**
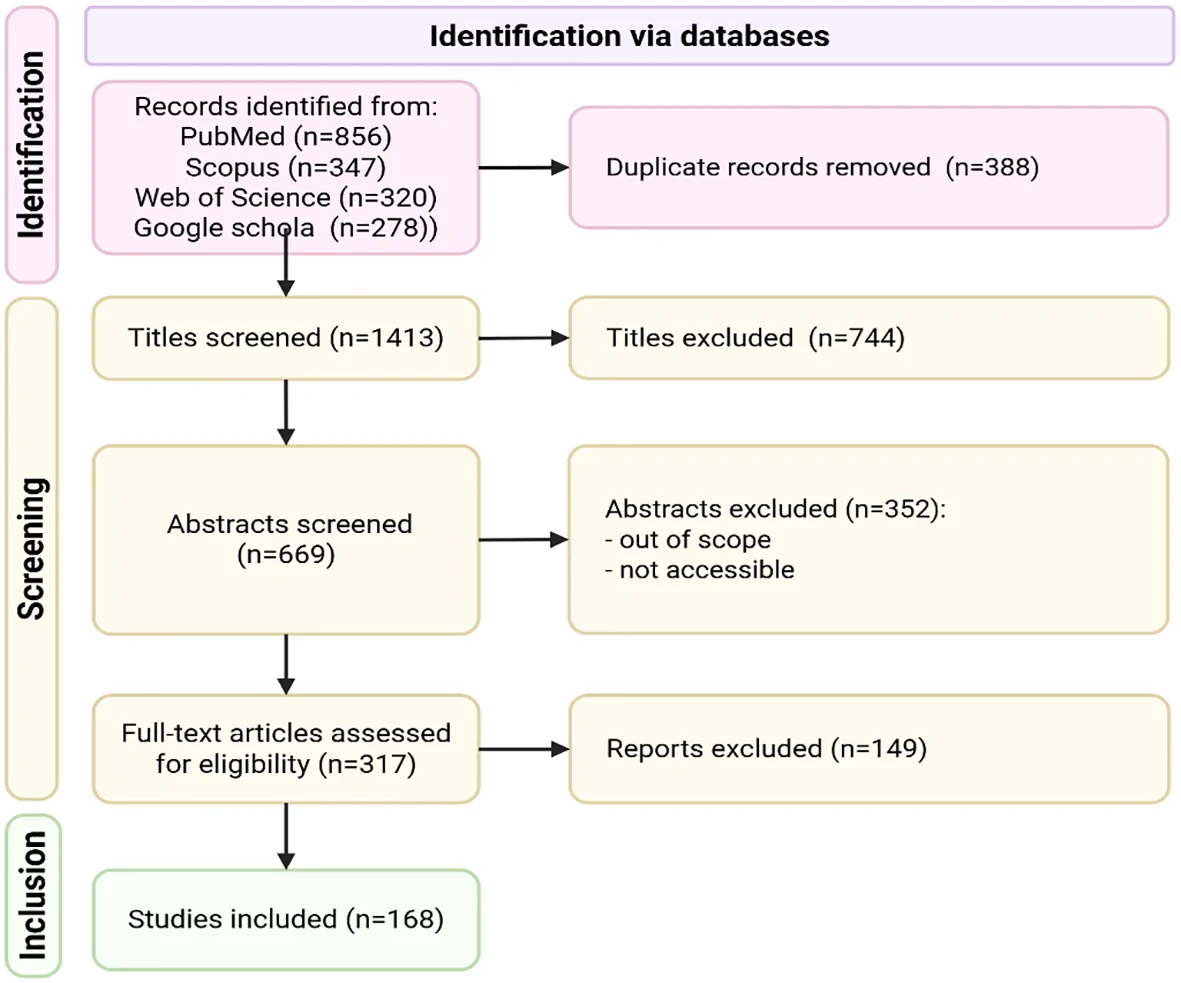
PRISMA-based flow diagram illustrating the literature identification and screening process (Rethlefsen and Page, 2022).

A structured literature search was conducted to identify studies related to amylose biosynthesis, genetic regulation of starch composition, and the nutritional and metabolic implications of rice starch variation (**Figure 1**). Bibliographic databases including PubMed, Scopus, Web of Science, and Google Scholar were systematically searched using combinations of keywords associated with rice starch biology, genetic regulation, breeding strategies, and metabolic outcomes. The search strategy included terms such as “rice amylose,” “Waxy gene (Wx),” “GBSSI,” “starch biosynthesis,” “amylopectin structure,” “resistant starch,” “glycemic index,” “marker-assisted selection,” “genome editing,” and “CRISPR rice,” along with additional keywords related to environmental regulation and grain quality traits, including “grain filling temperature,” “starch branching enzyme,” “SSIIa,” “SBEIIb,” and “rice starch digestibility.” The search covered studies published between 2000 and 2026. A total of 1,801 records were identified across databases (PubMed, *n* = 856; Scopus, *n* = 347; Web of Science, *n* = 320; Google Scholar, *n* = 278). After removal of duplicate records (*n* = 388), 1,413 titles were screened, of which 744 were excluded based on relevance. Subsequently, 669 abstracts were assessed, and 352 records were excluded due to being outside the scope of the review or not accessible. A total of 317 full-text articles were evaluated for eligibility, and 149 reports were excluded at this stage. Ultimately, 168 studies were included in the final analysis.

## 3 Molecular regulation of amylose biosynthesis

**Figure 2:**
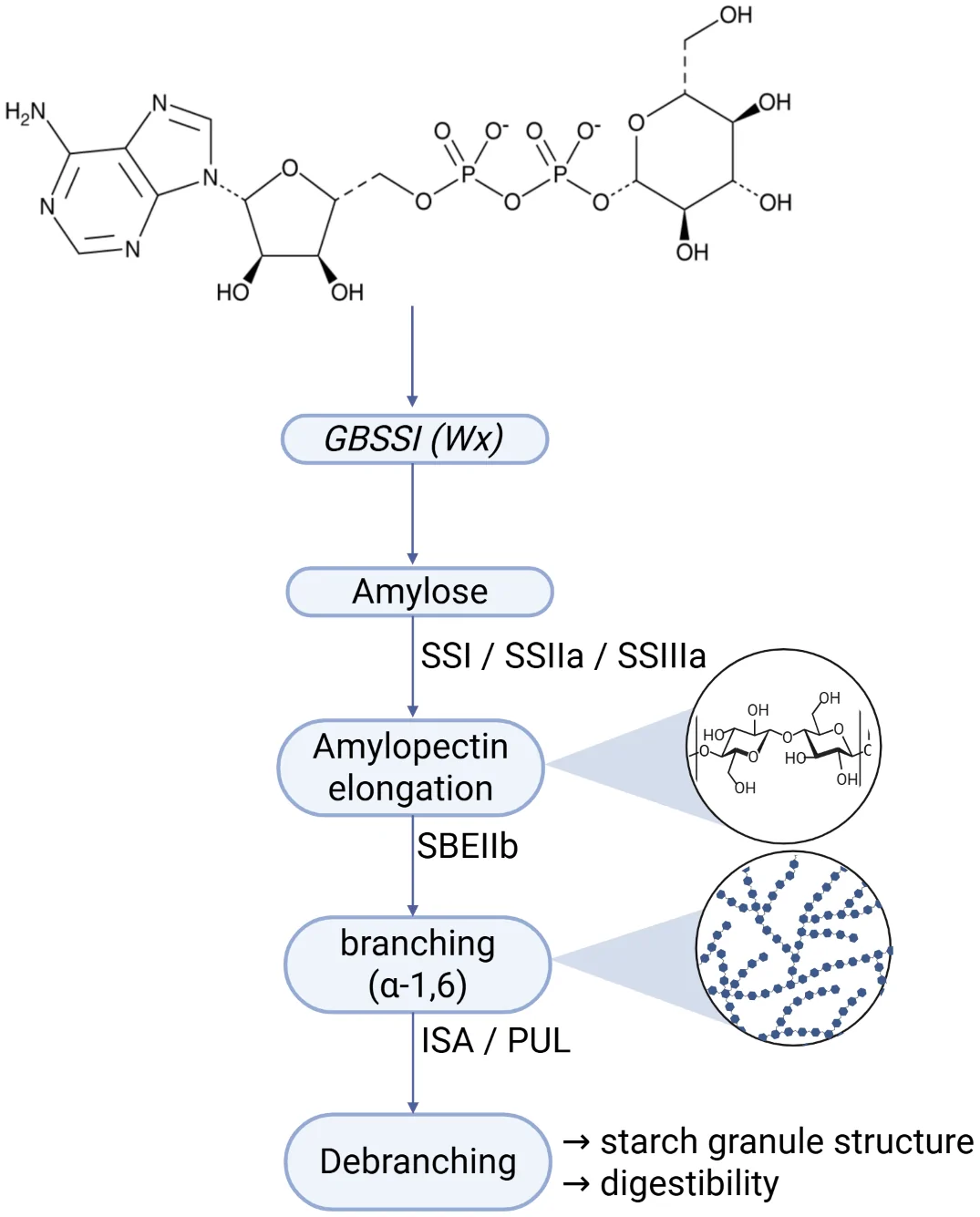
Molecular network regulating starch biosynthesis in rice endosperm. Starch biosynthesis begins with ADP-glucose and proceeds through the coordinated activities of starch synthases, starch branching enzymes (SBEs), and debranching enzymes (DBEs). Granule-bound starch synthase I (GBSSI), encoded by the *Wx* gene, catalyzes amylose synthesis, whereas soluble starch synthases primarily elongate amylopectin chains. SBEs introduce α-1,6 branch points, and DBEs remodel glucan chains to facilitate proper starch granule formation. The coordinated activities of these enzymes determine amylose and amylopectin composition, starch granule architecture, and, ultimately, rice grain quality and digestibility (Sharma and Khanna, 2019).

Amylose biosynthesis in rice endosperm is regulated by a coordinated enzymatic network that controls glucan elongation, branching, and debranching, thereby determining the relative accumulation of amylose and amylopectin as well as the structural organization of starch granules (**Figure 2**) (Sharma and Khanna, 2019).

### 3.1 Structural basis of starch functionality

Rice starch functionality is governed by a hierarchical structural framework in which molecular composition, supramolecular organization, and granule architecture collectively determine thermal behavior, processing performance, and digestibility (Wang et al., 2020). In rice endosperm, starch biosynthesis produces two principal polymers, amylose and amylopectin, from ADP-glucose. Amylose consists of primarily linear α-1,4-linked glucan chains synthesized by granule-bound starch synthase I (GBSSI), whereas amylopectin forms a highly branched polymer generated through the coordinated activity of starch synthases and branching enzymes (Li and Gilbert, 2016; Li et al., 2016; Tao et al., 2019). Variation in amylose proportion and amylopectin chain-length distribution strongly influences the crystalline structure and functional properties, with longer amylopectin chains generally associated with increased structural order and altered gelatinization and textural behavior (Zaman et al., 2025). These features vary in a coordinated manner across rice germplasm, indicating that starch functionality reflects integrated structural parameters rather than a single compositional trait (Zhang et al., 2023).

This structural organization is largely determined by the starch biosynthetic machinery, particularly enzymes controlling amylopectin branching. Starch branching enzyme IIb (SBEIIb) plays a central role in defining chain distribution, and its downregulation leads to reduced branching, increased long-chain content, and a shift toward high resistant starch (RS) phenotypes (Vandeputte and Delcour, 2004; Butardo et al., 2011; Tetlow and Emes, 2014). Notably, even moderate changes in SBEIIb expression can significantly alter starch structure and digestibility, demonstrating that relatively small modifications in branching patterns can produce measurable functional effects without disrupting overall starch synthesis (Yang et al., 2026).

At the physicochemical level, these structural differences are expressed through gelatinization, swelling, paste viscosity, and retrogradation behavior. Gelatinization reflects the disruption of crystalline regions during heating, whereas retrogradation corresponds to reassociation of starch chains during cooling (Gong et al., 2024). Starches with higher structural order exhibit greater thermal stability and lower swelling capacity, while less ordered structures gelatinize more readily (Li et al, 2022). In addition, interactions with non-starch components, particularly proteins and lipids, further modulate functional behavior in processed systems such as rice-based foods (Farooq and Yu, 2025). Consistent with this framework, grain quality traits in rice, including texture and eating quality, are closely associated with starch structural features such as viscosity profiles and gelatinization characteristics (Zhu et al., 2021).

Overall, starch functionality in rice is determined by the combined effects of amylose content, amylopectin architecture, and post-processing transformations (**Table 1**). Amylose promotes dense molecular packing, crystallinity, and retrogradation, thereby reducing enzymatic digestibility and glycemic response, whereas the amylopectin structure governs granule organization, gelatinization properties, and texture (Kaur et al., 2016; Li et al., 2016; Kumar et al., 2018a; Sharma and Khanna, 2019; Farooq and Yu, 2025). Importantly, these effects operate along a continuum rather than a binary system, as amylopectin fine structure, particularly chain length distribution and branching frequency, can significantly influence starch digestibility independently of amylose content (Kaur et al., 2016; Kumar et al., 2018a; Harris, 2019).

**Table 1:** Structural features of rice starch and their functional implications.

Structural feature	Molecular characteristics	Enzymatic/genetic control	Functional consequences	Ref.
Amylose content and structure	Linear α-1,4-glucan; MW 10^5^–10^6^ Da; DP 500–5,000; low branching (0.1–0.5%)	GBSSI (Wx gene)	Promotes crystallinity and double helix formation; increases firmness after cooking; enhances retrogradation and RS3 formation; lowers GI	(Kaur et al., 2016; Li et al., 2016; Kumar et al., 2018a; Farooq and Yu, 2025)
Amylopectin structure (branching pattern)	Highly branched polymer; MW >10^8^ Da; short chains (DP 6–12), long chains (DP 13–24); cluster organization	SS, SBE enzymes	Determines semi-crystalline structure; affects gelatinization temperature (GT), viscosity, and texture; influences digestibility and GI	(Li et al., 2016; Kumar et al., 2018a; Farooq and Yu, 2025)
Crystalline organization	Alternating crystalline and amorphous lamellae; B-type polymorphs (amylose-rich)	Chain-length distribution; hydrogen bonding	Controls water absorption and swelling; defines thermal stability and cooking properties	(Li et al., 2016; Farooq and Yu, 2025)
Gelatinization properties	Disruption of crystalline regions at 55 °C–80 °C	SSIIa (ALK gene) alleles	Determines cooking time, energy requirement, and texture of cooked rice	(Sharma and Khanna, 2019)
Retrogradation behavior	Reassociation of starch chains during cooling; stronger in high-amylose starch	Amylose content; chain interactions	Leads to firmness, staling, and formation of resistant starch type 3 (RS3)	(Kaur et al., 2016; Li et al., 2016)
Amylose–lipid complexes (RS5)	Inclusion complexes between amylose and lipids	Processing conditions (cooking)	Reduces enzymatic digestibility; contributes to lower GI	(Harris, 2019)
Digestibility and glycemic response	Enzyme accessibility influenced by crystallinity and branching	Integrated effect of GBSSI, SS, SBE	High amylose and longer amylopectin chains reduce digestion rate; lower GI	(Li et al., 2016; Kumar et al., 2018a; Harris, 2019)

GBSSI, granule-bound starch synthase I; Wx, Waxy gene; SS, starch synthase; SBE, starch branching enzyme; SSIIa, starch synthase IIa; ALK, Alkali degeneration gene; DP, degree of polymerization; MW, molecular weight; GT, gelatinization temperature; RS3, resistant starch type 3; RS5, resistant starch type 5; GI, glycemic index.

### 3.2 *Waxy* gene and functional alleles

**Figure 3:**
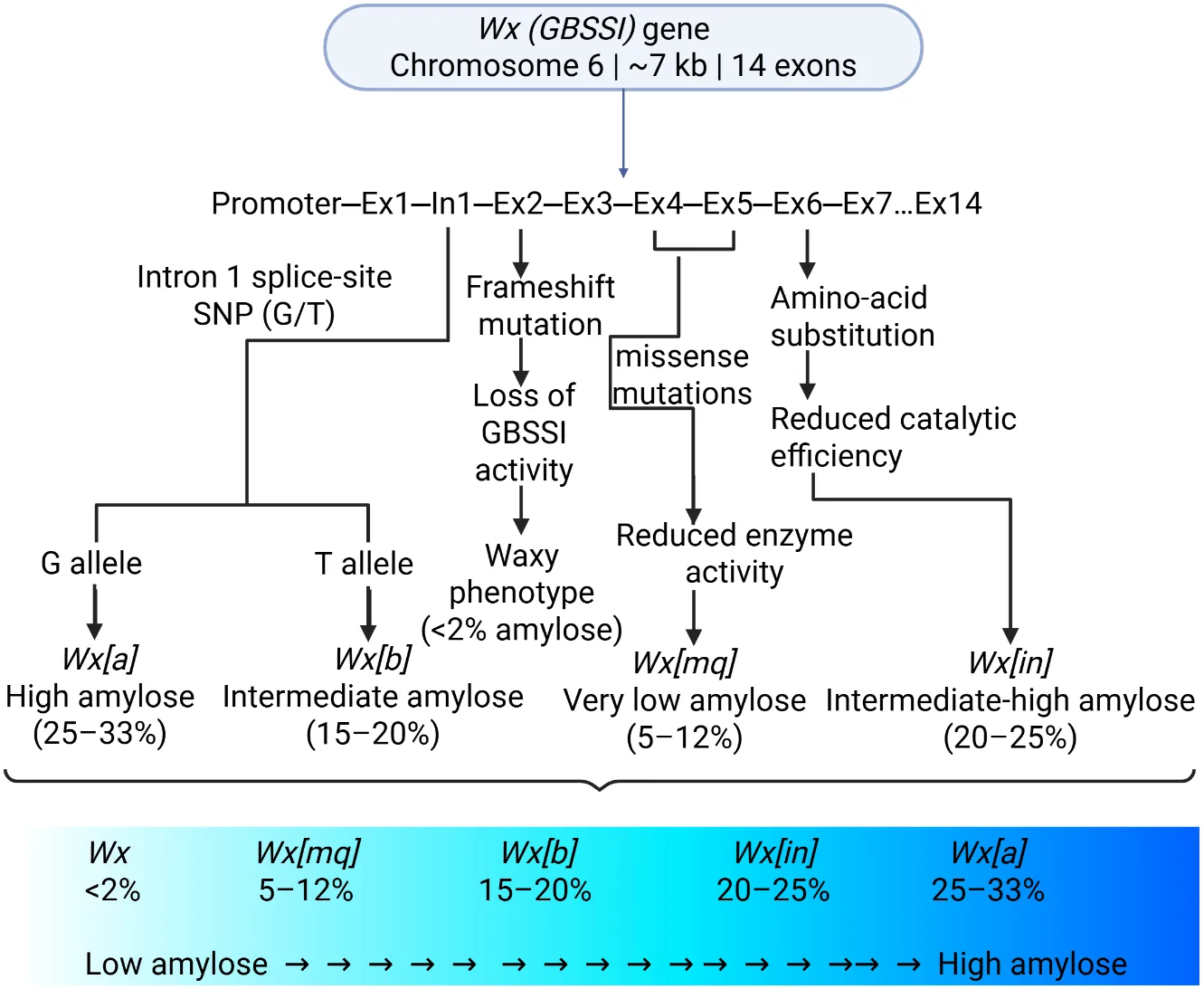
Structure of the *Wx* gene and major functional alleles controlling amylosecontent in rice. The *Wx* gene located on chromosome 6 encodes granule-bound starchsynthase I (GBSSI), the key enzyme responsible for amylose biosynthesis in rice endosperm. Natural polymorphisms within the *Wx* locus generate several functional alleles that determine variation in amylose content among rice cultivars. A G/T single-nucleotide polymorphism at the intron-1 splice donor site differentiates the high-amylose *Wx[a]* allele from the intermediate-amylose *Wx[b]* allele by altering pre-mRNA splicing efficiency. Additional mutations, including a 23-bp duplication in exon 2 resulting in the waxy (*wx*) phenotype, missense mutations in exons 4–5 (*Wx[mq]*), and a nucleotide substitution in exon 6 (*Wx[in]*), further modulate GBSSI activity and produce a gradient of amylose levels in rice grains (UniProt Consortium; Sharma and Khanna, 2019; Farooq and Yu, 2025).

The *Waxy* (*Wx*) gene represents the principal genetic determinant of AC in rice and plays a central role in regulating starch composition and grain quality (**Figure 3**). Located on chromosome 6, *Wx* encodes GBSSI, the enzyme responsible for elongating α-1,4-glucan chains during amylose biosynthesis within developing endosperm amyloplasts (Sharma and Khanna, 2019). The gene spans approximately 7 kb and comprises 14 exons encoding a 608-amino-acid protein harboring a plastid-targeting transit peptide that mediates localization to starch granules (UniProt Consortium; Farooq and Yu, 2025). Within the granule matrix, GBSSI catalyzes the incorporation of glucose residues from ADP-glucose into growing glucan chains, thereby generating the largely linear polymers that define the amylose structure (Zhou et al., 2015).

Natural variation at the *Wx* locus explains a substantial proportion of the diversity in AC observed among cultivated rice varieties. Three major allelic classes, *Wx[a]*, *Wx[b]*, and *wx*, account for most of this variation across *indica* and *japonica* subspecies (Sharma and Khanna, 2019). The *Wx[a]* allele, predominantly found in *indica* rice, contains a guanine nucleotide at the 5′ splice donor site of intron 1 that promotes efficient pre-mRNA splicing, resulting in high GBSSI expression and typically high amylose content, generally ranging from approximately 22% to 28%, although some genotypes may exhibit higher values (Larkin and Park, 2003). In contrast, the *Wx[b]* allele, characteristic of *japonica* rice, carries a thymine substitution at this splice site that reduces splicing efficiency and lowers GBSSI accumulation, resulting in intermediate amylose levels of approximately 15%–20% (Hoai et al., 2014; Kumar et al., 2018a). The recessive *wx* allele, associated with waxy or glutinous rice, usually contains a 23-bp duplication in exon 2 that causes a frameshift mutation and complete loss of GBSSI activity, producing starch composed almost entirely of amylopectin with amylose levels below 2% (Chrungoo and Devi, 2016).

Beyond these classical alleles, additional nucleotide polymorphisms within *Wx* further fine-tune amylose accumulation and contribute to the continuous spectrum of starch phenotypes observed in rice—for example, the *Wx[in]* allele carries a nucleotide substitution in exon 6 that alters the amino acid sequence of GBSSI and distinguishes high-amylose from intermediate-amylose *indica* cultivars (Shah et al., 2020). Similarly, the *Wx[mq]* allele identified in certain japonica varieties contains missense mutations in exons 4 and 5 that reduce enzymatic activity without markedly affecting gene expression, resulting in reduced AC and improved gel consistency (Yang et al., 2024). Regulatory variation also contributes to phenotypic diversity; promoter polymorphisms such as the *Wx[1764178]* allele influence transcriptional activity and modulate amylose levels under different nitrogen regimes (Tang et al., 2024).

Although AC broadly correlates with *Wx* transcription and GBSSI protein accumulation, the relationship between gene expression and starch phenotype is not strictly linear. Comparative analyses using near-isogenic lines indicate that transcript abundance generally follows the order *Wx[a] > Wx[b] > Wx[in] > wx*, which corresponds to decreasing AC (Zhang et al., 2012; Zhou et al., 2015). However, enzyme activity assays reveal that specific amino acid substitutions can alter catalytic efficiency independently of protein abundance, indicating that post-transcriptional and post-translational mechanisms also contribute to the regulation of amylose biosynthesis (Larkin and Park, 2003; Yang et al., 2024). These findings together highlight the *Wx* locus as a major but not solitary regulator of starch composition and emphasize the importance of both structural and regulatory variation in shaping rice grain quality.

### 3.3 Modifier genes in the starch biosynthesis network

Although the *Wx* gene provides the primary genetic control of AC, starch composition in rice is ultimately determined by a broader biosynthetic network involving multiple enzymes that regulate amylopectin architecture, granule formation, and starch crystallinity. A variation in these modifier genes can substantially influence apparent amylose content (AAC), RS formation, and cooking quality by altering the structural context in which amylose accumulates (Frei et al., 2003; Sharma and Khanna, 2019).

A central group of modifiers consists of starch branching enzymes that determine the frequency and distribution of α-1,6 branch points in amylopectin. Among them, starch branching enzyme IIb (SBEIIb), encoded on chromosome 2, plays a dominant role in generating short amylopectin branches with degrees of polymerization (DP) of approximately 6–12 (Busi et al., 2012). Suppression or knockout of *SBEIIb* markedly alters the amylopectin architecture by reducing branching density, which indirectly promotes higher apparent amylose levels and increased RS formation (Zhu et al., 2012; Sun et al., 2017). Functional studies, including CRISPR/Cas9-generated mutants, demonstrate that reduced *SBEIIb* activity leads to modified starch granule morphology, elevated gelatinization temperature, and improved glycemic characteristics of rice starch (Cong et al., 2013; Feng et al., 2014; Yamasaki et al., 2007; Sun et al., 2017; Gauda et al., 2023). These results highlight the importance of branching patterns in determining starch digestibility and nutritional properties.

A second group of modifiers includes elongating starch synthases that shape amylopectin chain-length distribution. Starch synthase IIa (SSIIa), encoded by the *ALK* gene on chromosome 6, extends intermediate amylopectin chains and plays a major role in determining gelatinization temperature and cooking behavior (Sharma and Khanna, 2019). Although SSIIa does not directly control amylose synthesis, allelic variation in *ALK* significantly modifies amylopectin structure and interacts epistatically with *Wx* alleles to influence overall grain quality (Farooq and Yu, 2025). Loss-of-function mutations in SSIIa alter amylopectin chain distribution and may indirectly increase AAC through structural compensation within the starch matrix (Busi et al., 2012).

Additional elongating enzymes, including starch synthase I (SSI) and starch synthase IIIa (SSIIIa), further contribute to amylopectin biosynthesis by extending short and long glucan chains, respectively (Farooq and Yu, 2025). Mutations in *SSIIIa* have been shown to increase both amylose and RS levels, reflecting the broader impact of amylopectin architecture on starch digestibility. Notably, combined mutations in *SSIIIa* and *SBEIIb* produce synergistic effects, leading to substantial increases in RS content and pronounced reductions in postprandial glycemic response (Tanaka et al., 2018; Saito et al., 2020). Environmental factors such as elevated temperature during grain filling can further amplify these effects by modifying starch structure and lipid interactions in developing grains (Zhou et al., 2022).

Debranching enzymes also play an essential role in shaping starch granule organization. Isoamylase (ISA) and pullulanase (PUL) hydrolyze specific α-1,6 linkages during amylopectin maturation, enabling the formation of ordered crystalline lamellae within starch granules (Farooq and Yu, 2025). Disruption of isoamylase activity, particularly mutations in *ISA1*, leads to the accumulation of highly branched phytoglycogen instead of normal starch, demonstrating the critical importance of controlled debranching for proper starch crystallization and granule formation (Zhou et al., 2022; Farooq and Yu, 2025).

Collectively, these studies demonstrate that amylose content and RS formation are not governed by a single gene but instead emerge from coordinated interactions among multiple enzymes in the starch biosynthetic pathway. Genetic analyses have identified combinations of single-nucleotide polymorphisms across several biosynthetic genes, including *GBSSI*, *SSIIa*, and *SSIIIa*, that jointly increase amylose and RS concentrations (Suzuki et al., 2008; Gurunathan et al., 2019). Systems genetics approaches further reveal complex epistatic relationships among these loci, indicating that starch composition is controlled by an integrated regulatory network rather than independent gene effects (Butardo et al., 2017). Understanding these interactions provides a foundation for breeding strategies that target multiple genes simultaneously to optimize starch structure, nutritional properties, and grain quality.

### 3.4 Environmental modulation of amylose biosynthesis

AC in rice is not determined solely by genetic variation but is strongly influenced by genotype × environment (G × E) interactions. Among environmental factors, temperature during the grain filling stage represents the most critical determinant of AC stability. Numerous studies show that high temperatures during endosperm development generally reduce AC, whereas cooler grain-filling conditions tend to increase amylose accumulation (Bao et al., 2004; Suzuki et al., 2008; Zhang et al., 2021). In practical terms, exposure to temperatures above approximately 30 °C during grain filling typically lowers AC by several percentage points, whereas cooler conditions below approximately 20 °C promote higher amylose levels. This environmental sensitivity introduces substantial variability in starch composition across production environments and therefore represents a major challenge for breeding programs targeting stable grain quality (Wang et al., 2017).

The molecular basis of this temperature response is closely linked to the regulation of the *Waxy* (*Wx*) gene. Elevated temperatures during grain filling have been shown to reduce *Wx* transcript abundance and GBSSI protein accumulation, particularly in genetic backgrounds carrying the *Wx[b]* allele, resulting in reduced amylose synthesis (Fan et al., 2023). In addition to transcriptional effects, temperature can influence the efficiency of *Wx* pre-mRNA splicing, further modulating GBSSI expression levels and contributing to variations in amylose accumulation.

Genetic modifiers that buffer the impact of environmental stress on *Wx* expression have therefore become important targets for breeding climate-resilient rice varieties. Several quantitative trait loci (QTLs), including qHAC4, qHAC8a, qHAC8b, and qHAC10, have been identified as regulators of amylose stability under high-temperature conditions (Zhang et al., 2014). These loci appear to mitigate temperature-induced reductions in AC by improving *Wx* pre-mRNA splicing efficiency or stabilizing gene expression during grain filling. Such QTLs represent valuable genetic resources for maintaining desirable starch composition in environments increasingly affected by heat stress.

Genetic background also modulates temperature sensitivity at the *Wx* locus. Comparative analyses between *Wx[a]* and *Wx[b]* alleles in *indica* and *japonica* genetic backgrounds demonstrate that the same allele can exhibit different responses to temperature depending on the surrounding genome (Fan et al., 2023)—for example, *Wx[a]* expression in certain *indica* cultivars is less stable under high temperature than in *japonica* near-isogenic lines carrying the same allele, indicating that additional modifier loci influence environmental responsiveness. Similarly, the *Wx[b]* allele exhibits higher amylose levels under cooler grain filling temperatures, suggesting that temperature-dependent regulation of starch biosynthesis involves multiple interacting genes (Suzuki et al., 2008).

Beyond structural gene variation, transcriptional regulators also contribute to the environmental modulation of amylose synthesis. The MADS-box transcription factor OsMADS7, originally characterized for its role in floral development, has also been implicated in rice responses to high temperature stress during grain filling. The experimental suppression of OsMADS7, particularly in the endosperm, has been associated with improved maintenance of amylose content and grain quality under heat stress. However, the available evidence suggests that this effect is likely mediated indirectly through the regulation of starch biosynthetic pathways rather than through direct regulation of *Wx* expression (Zhang et al., 2018). These findings indicate that manipulation of upstream regulatory networks may provide an alternative strategy for improving the stability of starch quality under elevated temperature conditions.

Additional genetic loci further contribute to environmental buffering of amylose levels—for example, the qSAC3 locus derived from *indica* germplasm has been shown to increase AC across multiple environmental conditions, highlighting the potential for incorporating stability alleles into breeding programs (Zhang et al., 2019). Taken together, these findings demonstrate that amylose phenotype stability emerges from complex interactions among *Wx* alleles, modifier genes, transcriptional regulators, and environmental conditions.

From a breeding perspective, the strong G × E interaction affecting amylose accumulation underscores the importance of evaluating candidate varieties across multiple environments. Environmental temperature can substantially alter starch composition, and therefore multi-location trials spanning diverse climatic conditions are essential for ensuring consistent grain quality in released cultivars (Wang et al., 2017; Zhang et al., 2019). Integrating genomic selection, climate-resilient alleles, and environment-specific evaluation will be critical for developing rice varieties that maintain desirable amylose levels under increasingly variable production conditions.

## 4 Classification of rice according to apparent amylose content

Apparent amylose content (AAC) is widely recognized as one of the most important determinants of rice grain quality because it strongly influences cooking behavior, texture, processing performance, and nutritional properties (Sharma and Khanna, 2019). Rice cultivars are therefore routinely classified into AAC categories despite amylose content being a continuous quantitative trait (Fu et al., 2022). This classification provides a practical basis for comparing cultivars with similar physicochemical characteristics and facilitates breeding for specific consumer preferences and end-use applications (Kaur et al., 2016). Each AAC class exhibits distinct physicochemical and nutritional properties that determine its suitability for specific culinary, industrial, and health-related applications (**Table 2**) (Zhang et al., 2018).

**Table 2:** Classification of rice according to AAC and associated functional characteristics.

Amylose class	AAC (%)	Representative genetic determinants	Major starch characteristics	Cooking and eating quality	Health and functional implications	Typical applications
Waxy	0–2	*wx* loss-of-function alleles (Sharma and Khanna, 2019; Fu et al., 2022)	Nearly amylose-free starch; amylopectin-dominant composition; high paste viscosity (Zhang et al., 2018; Fu et al., 2022)	Extremely sticky, translucent, soft cooked grains (Zhang et al., 2018; Fu et al., 2022; Yang et al., 2022)	Very high glycemic index (GI), minimal RS (Hu et al., 2004; Kaur et al., 2016)	Mochi, rice cakes, desserts, processed foods
Very low amylose	2–12	*Wx*mq and related weak *Wx* alleles (Yang et al., 2022; Yang et al., 2024)	Low amylose with soft gel formation (Yang et al., 2022; Yang et al., 2024)	Very soft, highly sticky cooked rice (Yang et al., 2022; Yang et al., 2024)	High digestibility and relatively high GI (Hu et al., 2004)	Baby foods, porridges, soft-textured rice products
Low amylose	12–20	*Wx*b (japonica) (Sharma and Khanna, 2019)	Moderate amylose; limited retrogradation (Sharma and Khanna, 2019)	Soft, moderately sticky, glossy grains (Sharma and Khanna, 2019)	Moderate GI and relatively low RS (Kaur et al., 2016)	Sushi rice and premium japonica table rice
Intermediate amylose	20–25	*Wx*b or *Wx*in (Sharma and Khanna, 2019; Selvaraj et al., 2021)	Balanced amylose–amylopectin composition (Sharma and Khanna, 2019; Selvaraj et al., 2021)	Fluffy grains with moderate firmness and low stickiness (Sharma and Khanna, 2019; Selvaraj et al., 2021)	Moderate postprandial glycemic response (Kaur et al., 2016)	General table rice preferred in many countries
High amylose	25–28	*Wx*^a^ (*indica*) (Butardo et al., 2019; Li et al., 2024)	High amylose promotes starch retrogradation and RS formation (Kumar et al., 2018a; Harris, 2019)	Firm, dry, well-separated grains (Butardo et al., 2011; Butardo et al., 2017; Butardo et al., 2019; Li et al., 2024)	Increased RS and lower GI (Kumar et al., 2018a; Harris, 2019)	Fried rice, parboiled rice, diabetic-friendly products
Very high amylose	>33	*SBEIIb* mutants/suppression or engineered starch biosynthesis (Zhu et al., 2012; Sun et al., 2017)	Very high amylose and RS accumulation (Zhu et al., 2012; Sun et al., 2017)	Very firm texture with limited palatability (Sun et al., 2017)	Very high RS and very low GI; potential functional food ingredient (Kumar et al., 2018a; Harris, 2019)	Functional foods and low-GI specialty products

Amylose variation influences not only rice texture but also its physicochemical and nutritional properties. High-amylose rice typically exhibits higher gelatinization temperature and stronger retrogradation because linear amylose chains readily form stable double helices and crystalline domains, which results in longer cooking times and increased firmness during storage after cooking (Li et al., 2016; Sharma and Khanna, 2019). In contrast, low-amylose and waxy rice show greater starch granule swelling and higher paste viscosity, producing the soft and cohesive textures preferred in many East Asian culinary traditions (Sharma and Khanna, 2019). From a nutritional perspective, amylose concentration is closely associated with starch digestibility and glycemic response. High-amylose rice generally contains greater amounts of RS, particularly retrograded starch (RS3), which resists enzymatic digestion in the small intestine and contributes to lower postprandial glucose responses (Kumar et al., 2018a; Harris, 2019). Waxy rice composed almost entirely of amylopectin is rapidly digested and typically exhibits the highest GI values (Hu et al., 2004). Although the relationship between amylose content and GI is influenced by factors such as amylopectin fine structure, lipid interactions, and processing conditions, higher amylose levels are generally associated with lower GI values, usually around 50–70 compared with values exceeding 90 in waxy rice (Fitzgerald et al., 2011; Kaur et al., 2016). RS content also increases along the amylose gradient, rising from less than 1% in waxy rice to approximately 5%–15% in high-amylose varieties, with implications for satiety, colonic fermentation, and metabolic regulation (Kumar et al., 2018a; Harris, 2019).

## 5 Physiological significance of amylose variation in rice grain development

### 5.1 Grain filling physiology and carbon partitioning

**Figure 4:**
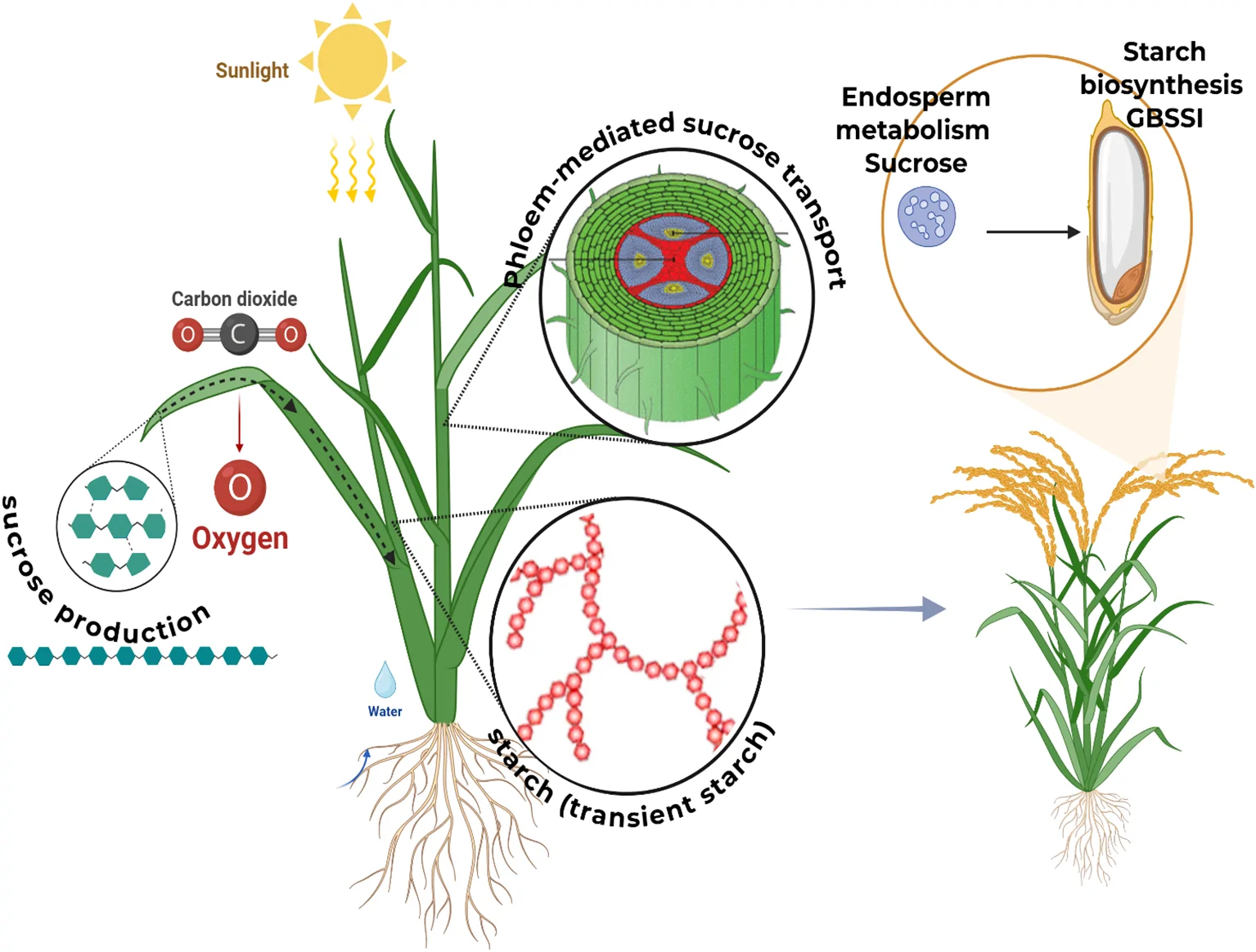
Source–sink carbon partitioning and starch biosynthesis during rice grain filling: carbon assimilated in photosynthetically active leaves is converted into sucrose and transported via the phloem to developing panicles. Part of the assimilates is transiently stored as starch in stem tissues and later remobilized as sucrose to sustain grain filling under fluctuating source activity. Upon arrival at the grain, sucrose is unloaded through the pericarp and nucellar projection and transported into the endosperm, where it is cleaved by sucrose synthase and invertases to generate UDP-glucose and glucose. UDP-glucose is subsequently converted into ADP-glucose, which serves as the substrate for starch biosynthesis in amyloplasts. Carbon partitioning within the endosperm is controlled by the coordinated activity of starch biosynthetic enzymes, where GBSSI directs amylose synthesis, while soluble starch synthases, branching enzymes, and debranching enzymes regulate amylopectin formation. The integration of source capacity, transport efficiency, and sink strength ultimately determines grain filling rate, starch accumulation, amylose content, and grain quality.

Grain filling in rice is governed by the coordinated movement of carbon from photosynthetically active source tissues to developing grains, where assimilates are converted into storage starch (**Figure 4**). Sucrose is the principal long-distance transport form of photoassimilates and is exported from mature leaves through the phloem toward developing panicles. In addition to current photosynthesis, rice plants can transiently store carbohydrates in stems and later remobilize these reserves to support grain filling, particularly when photosynthetic activity declines due to senescence or developmental progression (Xu, 2010). Therefore, grain filling capacity depends not only on leaf photosynthetic output but also on the efficiency of phloem loading, long-distance sucrose transport, and remobilization of stored reserves. Experimental enhancement of phloem sucrose loading through phloem-specific sucrose transporter expression increases assimilate flux to panicles, accelerates grain filling, and improves grain size and yield, indicating that source export capacity can represent a limiting step in carbon delivery to grains (Zhang et al., 2012). After reaching the developing caryopsis, sucrose is unloaded through maternal tissues and transferred across the pericarp and nucellar projection before entering endosperm storage cells. This maternal-to-filial transport step represents a key physiological control point, as impairments in transfer cell development or transport efficiency at the interface can restrict carbon flow even under adequate whole-plant assimilate supply (Xu, 2010). Within the endosperm, imported sucrose is metabolically converted into storage precursors that support starch accumulation, linking carbon import with sink metabolism (Xu, 2010; Wang et al., 2015).

The efficiency of grain filling is determined by the timing, rate, and duration of starch accumulation. Rice grain filling is generally divided into early initiation, rapid deposition, and late maturation phases. The most active period of starch accumulation occurs during the first several weeks after flowering, particularly between 7 and 28 days after flowering, when sink metabolic activity is at its highest (Hwang et al., 2005; Durbha et al., 2024). During this period, starch biosynthetic capacity within the endosperm increases in a coordinated manner, supporting the rapid conversion of imported carbon into storage starch. Enzymatic activities associated with starch formation follow a temporally regulated pattern, ensuring that substrate utilization and polymer assembly are synchronized with the changing demands of the developing grain (Li et al., 2010). These temporal dynamics demonstrate that starch accumulation is a developmentally regulated sink process rather than a passive response to carbon availability.

Physiological variation within the panicle further illustrates the importance of sink strength in grain filling. Superior spikelets, located in upper panicle positions and flowering earlier, usually initiate filling sooner and accumulate starch more rapidly than inferior spikelets. Inferior spikelets often show delayed sucrose unloading and lower early activities of sucrose synthase and AGPase, resulting in slower filling rates, incomplete starch deposition, and greater quality heterogeneity within the panicle (Wang et al., 2015). Hormonal regulation also contributes to this difference since elevated ethylene and abscisic acid levels in inferior spikelets can suppress sucrose synthase, AGPase, and starch synthase activities, thereby reducing sink activity and starch accumulation (Zhu et al., 2011). These findings indicate that grain filling efficiency depends on both carbon supply from source tissues and the metabolic capacity of individual grains to import and utilize assimilates.

Source capacity strongly influences the amount of carbon available for grain filling. Canopy photosynthesis provides most of the assimilates required for starch synthesis, while stem carbohydrate reserves buffer the system when current photosynthetic activity is limited (Xu, 2010). Nutrient management also affects carbon availability, as optimized nitrogen supply supports sustained leaf photosynthesis, improves carbohydrate accumulation in functional leaves and grains, enhances mid- and late-stage grain filling rates, and reduces chalkiness by improving the balance between source supply and sink demand (Guo et al., 2022; Song et al., 2025). In contrast, low irradiance reduces source capacity primarily by limiting photosynthetic carbon assimilation, leading to reduced grain filling rate and altered filling duration (Prathap et al., 2019; Li et al., 2022a). Under drought conditions, remobilization of stored carbohydrates from vegetative tissues may partially compensate for reduced photosynthesis; however, this compensation is limited when sink tissues are unable to efficiently unload and utilize incoming sucrose (Liu et al., 2023).

Sink strength determines how effectively developing grains attract and convert assimilates into starch. In rice endosperm, sink strength depends on sucrose unloading capacity, sucrose-cleaving enzyme activity, ADP-glucose supply, and the coordinated function of starch biosynthetic processes, including starch synthases, branching enzymes, debranching enzymes, and granule-bound starch synthesis activity (Wang et al., 2015). Carbon partitioning between amylose and amylopectin is therefore an integral component of sink function. Amylose synthesis occurs within starch granules as part of the overall starch assembly process, while other enzymatic pathways contribute to amylopectin formation and structural organization of starch. Pulse-labeling studies have shown that carbon incorporation into amylose and amylopectin changes across ripening stages, indicating that partitioning between these starch fractions is developmentally dynamic rather than constant (Liu et al., 2023). Source–sink manipulations such as spikelet thinning or altered photosynthetic supply can modify starch biosynthetic activity, starch structure, and amylose content, demonstrating feedback regulation between carbon availability and starch composition (Okawa et al., 2003; Liu et al., 2023).

Grain filling physiology links whole-plant carbon assimilation with endosperm starch biosynthesis. Starch accumulation is limited by source photosynthetic capacity, phloem transport, sucrose unloading, ADP-glucose supply, sink strength, enzyme coordination, and the duration of active filling. Environmental factors such as light, drought, temperature, and nutrient status modify these processes by altering photosynthesis, hormone balance, enzyme activity, and source–sink relationships (Prathap et al., 2019; Li et al., 2022a). From a crop physiology and breeding perspective, stable amylose accumulation cannot be understood only as a molecular trait controlled by *Wx* or other starch biosynthetic genes. It should also be viewed as the outcome of integrated carbon partitioning during grain development, where source capacity and sink strength jointly determine starch quantity, starch structure, and final grain quality.

### 5.2 Temperature stress and amylose stability

Temperature during grain filling is one of the major environmental determinants of amylose stability in rice. High temperature, particularly during the mid to late stages of endosperm development, commonly reduces amylose content and alters the amylose-to-total-starch ratio. Controlled-environment and field studies show that exposure to approximately 30 °C–35 °C or higher during grain filling often lowers final amylose accumulation compared with moderate temperatures of approximately 22 °C–2°C, although the magnitude of reduction varies across genotypes (Cheng et al., 2005; Cen et al., 2018). This reduction in amylose is associated with impaired starch biosynthetic capacity during grain filling, leading to reduced efficiency of amylose formation during the period of active starch deposition (Cheng et al., 2005; Mondal et al., 2025).

The effect of high temperature on amylose accumulation reflects a disruption of coordinated carbon partitioning during grain filling. Heat stress reduces the overall efficiency of carbon assimilation and transport, leading to a decreased supply of substrates required for starch biosynthesis in developing grains. At the sink level, heat stress impairs the metabolic conversion of imported sucrose into starch, resulting in reduced starch deposition and altered carbohydrate accumulation patterns (Mondal et al., 2025). Consequently, soluble sugars may accumulate while starch synthesis declines, indicating inefficient utilization of imported carbon. These changes collectively contribute to a shift in carbon allocation away from amylose formation and a reduction in the amylose-to-amylopectin balance under elevated temperature conditions (Cheng et al., 2005).

High temperature also modifies the rate and duration of grain filling. In early grain development, elevated temperature may transiently accelerate metabolic activity and increase soluble sugar levels, but this is often followed by a premature decline of starch biosynthetic enzyme activity during middle and late filling. Consequently, the effective filling period becomes shorter, starch deposition terminates earlier, and final grain weight and starch content are reduced (Shao et al., 2021; Mondal et al., 2025). These changes are also associated with higher grain chalkiness, reflecting disrupted starch granule development and looser endosperm packing under heat stress (Yang et al., 2023). Combined heat and drought stress can intensify these effects by simultaneously limiting source photosynthesis and impairing sink metabolic capacity, resulting in stronger reductions in starch accumulation and amylose proportion than either stress alone (Mondal et al., 2025).

Carbon flux during grain filling is highly sensitive to heat stress, which disrupts the balance between carbon production, translocation, and utilization. Elevated temperature reduces the efficiency of assimilate transport to developing grains and shortens the effective grain filling period, thereby limiting starch accumulation and increasing chalkiness (Cui et al., 2010; Shao et al., 2021; Lu et al., 2024). The magnitude of these effects depends on the timing and duration of heat exposure, with reproductive stage and post-flowering stress having particularly strong impacts on dry matter translocation and grain filling performance (Cui et al., 2010; Chaturvedi et al., 2017). High night temperature can further aggravate these responses by reducing carbohydrate remobilization and altering biomass partitioning, especially under suboptimal nitrogen availability (Lu et al., 2024). Although remobilization of pre-anthesis stem reserves may partially sustain grain filling under heat stress, its contribution is often insufficient to fully compensate for reduced assimilate transport and utilization in developing grains (Cui et al., 2010; Mondal et al., 2025).

Overall, temperature stress affects amylose stability through an integrated network involving carbon assimilation, assimilate transport, sink metabolic activity, and coordinated starch biosynthetic processes. Genotypic differences in these processes explain why some varieties maintain relatively stable amylose content and grain quality under heat stress, whereas others show pronounced reductions in amylose accumulation and increased chalkiness (Zhao et al., 2019; Yang et al., 2023; Dou et al., 2024). From a crop physiology and breeding perspective, temperature-resilient amylose accumulation should therefore be viewed as a whole-plant trait shaped by the coordination of carbon supply, transport efficiency, and sink utilization capacity during grain filling.

### 5.3 Amylose-related grain quality and crop adaptation

Amylose content is a major determinant of rice grain quality because it directly influences cooked texture, processing performance, nutritional properties, and consumer acceptance. High-amylose rice generally produces firmer, less sticky cooked grains, whereas low-amylose and waxy rice produce softer and more adhesive textures. Intermediate-amylose rice typically shows balanced eating quality, which explains its wide acceptance across markets (Chen et al., 2021). Although *Wx* is the primary genetic determinant of amylose accumulation, allelic variation at loci such as *SSIIa* and *SBEIIb* modifies grain quality by altering starch structure and functionality, resulting in distinct quality profiles even at similar amylose levels (Naseer et al., 2021). In breeding practice, amylose content is closely associated with key functional traits such as pasting properties and cooking behavior. Rapid Visco Analyzer parameters, particularly setback and final viscosity, are widely used as indirect indicators of amylose level and cooked rice texture (Oko et al., 2012; Hu et al., 2022). However, accurate prediction of grain quality requires multi-trait evaluation since amylose effects are modulated by amylopectin structure, protein content, lipids, and grain morphology (Gong et al., 2022; Hu et al., 2022).

Amylose content also plays a central role in determining nutritional quality. High-amylose rice is generally associated with increased RS formation and lower starch digestibility, resulting in reduced postprandial glycemic response (Chen et al., 2021; Lee et al., 2025). Nevertheless, GI is not controlled by amylose alone, as amylopectin fine structure, grain composition, and processing methods significantly modify digestibility (Huang et al., 2021; Lee et al., 2025). Post-cooking treatments such as cooling and parboiling further influence RS formation, which means that final nutritional outcomes depend on both genotype and processing conditions (Parween et al., 2020; Teixeira et al., 2025).

From a crop adaptation perspective, amylose content must be aligned with regional consumer preferences and production systems. Low- to intermediate-amylose rice is preferred in East and Southeast Asia due to its softer texture, whereas higher-amylose rice is favored in South Asia for its firmer and non-sticky grains (Oko et al., 2012). Therefore, optimal amylose levels are market-specific, and breeding programs must target defined quality classes rather than a universal standard. Haplotype-based selection at *Wx*, *SSIIa*, and *SBEIIb* enables the development of varieties combining desirable texture, nutritional properties, and cooking performance (Selvaraj et al., 2021).

Modification of amylose content is frequently associated with trade-offs affecting yield, grain appearance, and processing quality. Increased RS or altered amylose-to-amylopectin balance can reduce grain weight, increase chalkiness, and disrupt endosperm structure (Parween et al., 2020; Miura et al., 2022). While higher amylose may improve grain integrity in some processing applications, excessive levels can negatively affect sensory quality and post-cooking texture stability (Parween et al., 2020; Zapater et al., 2023). These trade-offs highlight the need to balance amylose improvement with agronomic performance and consumer preferences.

For elite cultivar development, amylose content should be selected together with complementary traits, including gel consistency, gelatinization temperature, RVA profile, RS content, chalkiness, and grain shape. MAS targeting *Wx*, *SSIIa*, and *SBEIIb* accelerates the optimization of these traits, while multi-environment testing is essential to ensure the stability of grain quality under variable field conditions (Cen et al., 2018; Guo et al., 2022). Thus, amylose content should be considered not only as a biochemical parameter but also as an integrative trait linking grain quality, nutrition, and crop adaptation.

## 6 Rice germplasm resources for grain quality improvement

### 6.1 Natural variation across *indica*, *japonica*, and waxy rice

**Figure 5:**
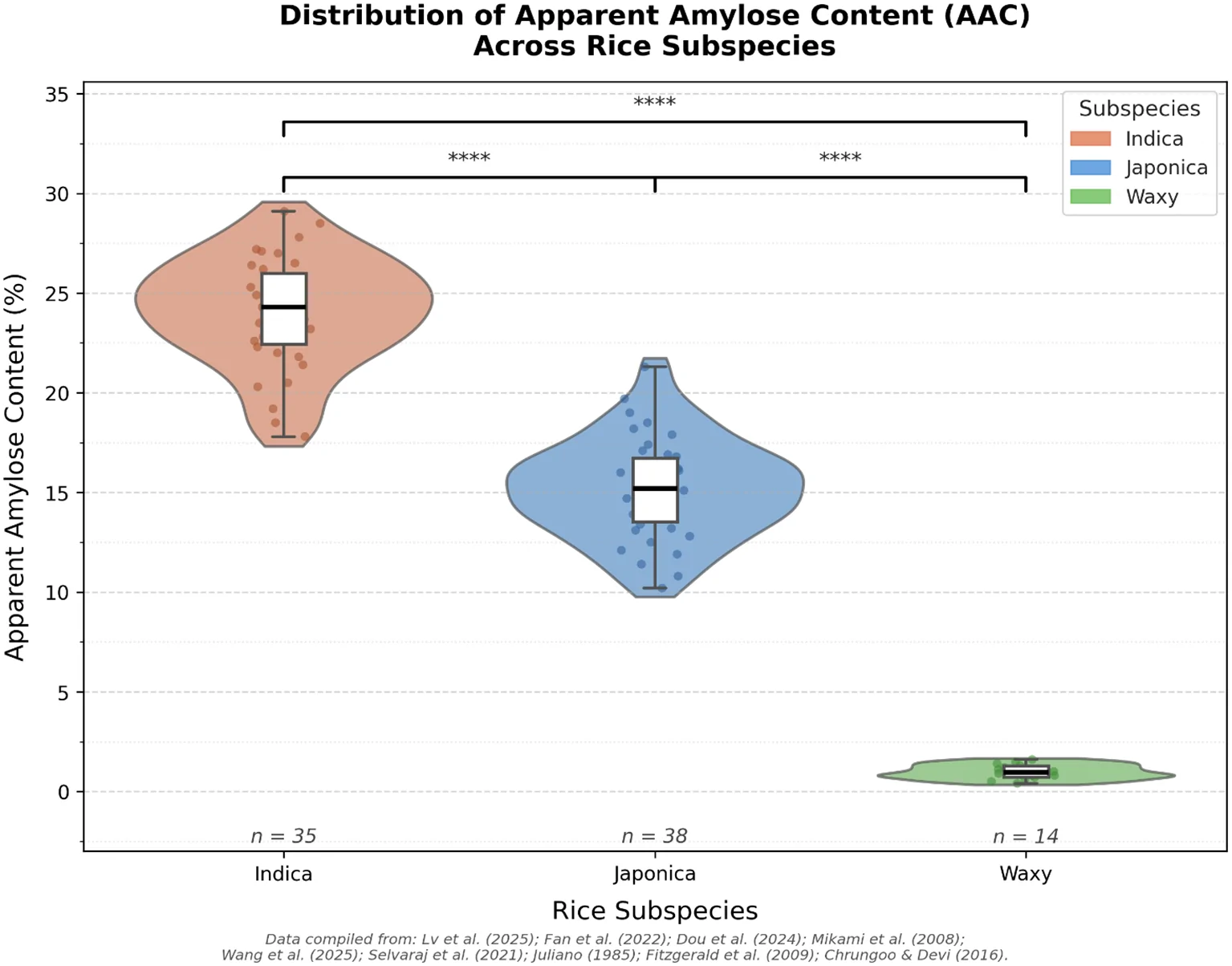
Subspecies-specific variation in AAC across rice germplasm: Violin plots with embedded boxplots showing AAC (%) distributions in indica, japonica, and waxy rice. Indica exhibits significantly higher AAC (~23% to 24%), japonica intermediate levels (~13%–15%), and waxy rice near-zero values. Statistical differences between groups are highly significant (****p < 0.0001). Sample sizes are indicated. Data compiled from (He et al., 2006; Li et al., 2006; Mikami et al., 2008; Zhao et al., 2010; Fitzgerald et al., 2011; Luo et al., 2015; Kharshiing et al., 2021; Selvaraj et al., 2021; Fan et al., 2023; Dou et al., 2024; Lv et al., 2025; Wang et al., 2025).

Natural variation in AAC is widely observed across rice germplasm and represents an important resource for grain-quality improvement (**Table 3**). Representative germplasm collections include elite indica and japonica breeding panels, regional landrace collections, and diverse global rice accessions that collectively encompass a broad spectrum of amylose phenotypes—for example, elite indica germplasm generally exhibits higher AAC than elite japonica germplasm, whereas regional collections from Yunnan and Northeast India demonstrate substantial within-population variation. Large diversity panels comprising hundreds of *O. sativa* accessions further illustrate the extensive phenotypic diversity present across cultivated rice populations (He et al., 2006; Li et al., 2006; Mikami et al., 2008; Luo et al., 2015; Kharshiing et al., 2021; Lv et al., 2025; Wang et al., 2025). These germplasm resources provide breeders with well-characterized donor materials for improving cooking quality, processing characteristics, and nutritional properties while also serving as valuable populations for genome-wide association studies, quantitative trait locus mapping, and pre-breeding programs. The distribution of AAC across major rice groups is summarized in **Figure 5**, whereas representative germplasm resources and their breeding value are presented in **Table 3**.

**Table 3:** Representative rice germplasm resources for AAC improvement and their characteristics.

Rice group	Typical AAC (%)	Representative varieties/germplasm resources	Geographic origin	Major grain quality characteristics	Typical end-use	Breeding relevance	Ref.
Indica	1.5–26.4 (mean 17.47)	*IR64*, *Minghui 63*, *Teqing*; elite indica panel (*n* = 152); representative accessions from Guangdong and Guizhou	Southern China; South and Southeast Asia	High AAC, firm cooked texture, relatively low stickiness, higher protein content	Table rice, parboiled rice	Widely used donor germplasm for improving amylose content, grain quality, and processing performance	(Li et al., 2006; Wang et al., 2025)
Japonica	0.7–24.6 (mean 15.76)	*Nipponbare*, *Koshihikari*, *Nanjing 42*, *Chujing 42*, *Xiangnuo 105*; elite japonica panel (*n* = 163)	Northern China, Japan, Korea, Yunnan	Lower AAC, soft texture, superior eating quality, higher head rice yield	Premium table rice, sushi	Important donor germplasm for improving eating quality, appearance, and milling quality	(Li et al., 2006; Wang et al., 2025)
Yunnan local germplasm	Overall mean 21.70 (range 3.74–29.84)	201 local accessions (175 *Wxa*, 20 *Wxb*, 4 *Wxin*, 2 *Wxmw*)	Yunnan Province, China	Predominantly indica-type germplasm with broad amylose diversity and extensive phenotypic variation	Local and regional rice production	Valuable landrace resource for germplasm conservation, pre-breeding, and marker-assisted selection	(Lv et al., 2025)
Asian rice landraces	0.5–29.9	Global diversity panel of 395 *O. sativa* accessions representing indica, aus, temperate japonica, tropical japonica, aromatic (group V), admixed germplasm, and representative landraces carrying *Wxa*, *Wxin*, *Wxb*, *Wxop*, and *wx* alleles	Asia (China, Japan, Taiwan, India, Indonesia, and other rice-growing regions)	Broad natural variation in amylose content associated with diverse *Wx* haplotypes and population structure	Diverse traditional food products	Excellent resource for allele mining, association mapping, population genomics, and marker-assisted breeding	(Mikami et al., 2008; Luo et al., 2015)
Northeast Indian rice germplasm	1.38–28.48	Thirty-five accessions (18 indica, 17 japonica) representing six *Wx* alleles (*wx*, *Wxop*, *Wxb*, *Wxin*, *Wxa2*, and *Wxa1*)	Northeast India	Continuous variation from glutinous to high-amylose rice with contrasting cooking quality	Traditional and regional rice cultivation	Valuable germplasm for discovering novel functional alleles and developing molecular markers for starch-quality breeding	(Kharshiing et al., 2021)
Breeding populations and elite mapping germplasm	Variable (DH: 6.6–25.7; RIL: ~11–35)	Elite breeding panel (315 accessions: 163 japonica, 152 indica); DH population (*Nanjing11 × Balilla*, *n* = 130); F_2_ population (*Yangfunuo 4 × Suyunuo*, *n* = 501); RIL population (*IR64 × Nipponbare*)	China; Australia	Broad phenotypic variation in amylose content, cooking quality, and starch physicochemical properties	Commercial breeding and research	Reference populations for GWAS, QTL validation, marker-assisted selection, and evaluation of multi-gene effects on grain quality	(He et al., 2006; Li et al., 2006; Zhao et al., 2010; Luo et al., 2015; Wang et al., 2025)

Variation in AAC across rice germplasm reflects long-term domestication together with regional selection for contrasting culinary preferences. Comparative analyses of Chinese breeding germplasm showed that elite *japonica* cultivars from Jiangsu and Yunnan generally possess lower AAC, higher head rice yield, and superior eating quality, whereas elite *indica* cultivars from Guangdong and Guizhou exhibit higher AAC and firmer grain texture, illustrating how modern breeding has reinforced regional quality preferences (Li et al., 2006; Wang et al., 2025). Genome-wide analyses of a global diversity panel comprising 395 *O. sativa* accessions likewise identified five major genetic groups (indica, aus, temperate japonica, tropical japonica, and aromatic/group V) together with admixed accessions, highlighting the roles of domestication, adaptation, and introgression in shaping rice diversity (Luo et al., 2015). Studies of Asian landraces further demonstrated a continuous spectrum of amylose phenotypes (0.5–29.9%) associated with diverse *Wx* haplotypes, emphasizing the value of traditional germplasm as a reservoir of genetic diversity for grain quality improvement (Mikami et al., 2008). Similar patterns were observed in Yunnan local germplasm and Northeast Indian rice collections, where broad variation in amylose content was documented across representative accessions carrying multiple naturally occurring *Wx* alleles (Kharshiing et al., 2021; Lv et al., 2025).

These historical selection patterns closely correspond to regional consumer preferences. Rice with low or intermediate AAC is generally preferred in East Asian countries, including China, Japan, and Korea, because of its softer, stickier texture, whereas populations in South Asia commonly prefer higher-AAC rice with firmer, separate cooked grains (Zhao et al., 2010; Pokhrel et al., 2020). Nevertheless, amylose content should not be regarded as a fixed property of any germplasm group because environmental conditions during grain filling, particularly temperature, can substantially modify the final phenotype even within the same genotype (Dou et al., 2024). Therefore, germplasm evaluation across multiple environments remains essential for identifying stable donor materials for breeding. Collectively, the diversity represented by elite cultivars, regional landraces, global diversity panels, and experimental mapping populations provides a valuable foundation for marker-assisted selection, QTL validation, and the development of rice varieties with improved grain quality, processing performance, and nutritional characteristics (He et al., 2006; Li et al., 2006; Zhao et al., 2010; Luo et al., 2015; Wang et al., 2025).

### 6.2 Landraces and traditional varieties as sources of genetic diversity

Landraces and traditional rice varieties represent an important reservoir of diversity for grain-quality improvement because they have been maintained for generations under local farming systems rather than intensive modern breeding. Unlike elite cultivars, which were developed from a relatively narrow genetic base to achieve uniform agronomic performance, traditional germplasm has retained broad phenotypic variation shaped by farmer selection, local culinary preferences, and adaptation to diverse production environments. This diversity is reflected not only in AAC but also in cooking quality, grain texture, gel consistency, milling performance, nutritional composition, and starch digestibility—for example, Nepalese landraces exhibited exceptionally wide variation in AAC (7.23–58.39%), with the first four principal components explaining 73.8% of the observed phenotypic diversity, highlighting their value as potential donor materials for quality improvement (Pokhrel et al., 2020). Similarly, analysis of 706 Chinese indica landraces demonstrated substantially higher median amylose content and slower predicted starch digestibility than recently released cultivars, indicating that traditional germplasm preserves quality attributes that have been reduced during modern breeding (Huang et al., 2022). Comparable patterns have been reported for traditional rice collections from India and Malaysia, where significant differences were observed across multiple grain quality traits, including amylose content, gel consistency, grain morphology, and cooking characteristics, emphasizing that landraces provide a broad resource for breeding diverse market classes rather than a single desirable phenotype (Sarker and Roy, 2023; Abd Rahman et al., 2024). Collectively, these findings indicate that the principal value of landraces lies in the breadth of their phenotypic diversity, making them indispensable donor resources for broadening the genetic base of modern rice improvement programs while preserving grain quality traits that may otherwise be lost through selection.

Evidence from geographically distinct rice-growing regions consistently demonstrates that traditional landraces harbor substantially broader phenotypic diversity than is typically found in modern breeding materials. In Nepal, an evaluation of 30 traditional landraces revealed highly significant variation across physicochemical and cooking-quality traits, with apparent amylose content (AAC) ranging from 7.23% to 58.39% and the first four principal components explaining 73.8% of the total phenotypic variation, indicating the presence of multiple contrasting quality types within a relatively small collection (Pokhrel et al., 2020). Similar trends were observed in southern China, where an analysis of 706 indica landraces showed significantly higher median AAC (26.3%) and markedly slower predicted starch digestion than 1,836 recently released cultivars (*p* < 0.001), suggesting that modern breeding has narrowed part of the quality diversity preserved in traditional germplasm (Huang et al., 2022). Studies of indigenous rice from West Bengal and Malaysian landraces likewise reported significant differences in amylose content, gel consistency, grain morphology, and cooking characteristics, with broad-sense heritability for amylose content reaching 99.57% in the Malaysian collection, highlighting the potential of these materials as reliable donor resources for quality improvement (Sarker and Roy, 2023; Abd Rahman et al., 2024).

Comparable patterns have also been reported outside the major Asian breeding centers. Aromatic black rice landraces from northeastern India displayed extensive variation in amylose content (0.92%–13.56%), resistant starch (4.72%–21.17%), anthocyanin concentration, and cooking quality, demonstrating that nutritional and eating-quality traits can be simultaneously improved using traditional germplasm (Idrishi et al., 2024). The characterization of 215 *Oryza glaberrima* accessions likewise identified distinct groups combining high amylose content, reduced chalkiness, favorable milling performance, and desirable cooking properties, emphasizing the value of African rice as an additional source of grain-quality diversity beyond *O. sativa* (Gnacadja et al., 2022). Studies of traditional Nigerian rice further showed that several local cultivars matched or exceeded imported varieties in cooking performance, exhibiting favorable grain expansion, water uptake, and gel consistency while maintaining amylose contents of 18.3%–27.2% (Azuka et al., 2020). Collectively, these independent studies indicate that landraces maintained under diverse agroecological conditions consistently preserve broad variation in amylose content, cooking quality, starch digestibility, and adaptation-related traits. At the same time, most investigations remain limited to regional germplasm collections and phenotypic characterization, emphasizing the need to integrate these valuable donor resources into larger breeding populations and multi-environment validation programs (Azuka et al., 2020; Pokhrel et al., 2020; Zhao et al., 2020; Gnacadja et al., 2022; Huang et al., 2022; Sarker and Roy, 2023; Abd Rahman et al., 2024; Idrishi et al., 2024).

Traditional rice cultivation has also played a central role in conserving genetic diversity and promoting local adaptation under diverse agroecological conditions. Large-scale analyses of worldwide germplasm collections demonstrated that representative core collections can preserve most of the phenotypic, geographic, and genetic diversity present in thousands of traditional accessions while substantially reducing the population size required for breeding and genetic analyses, thereby facilitating the efficient utilization of landrace resources (Mogga et al., 2018; Huggins et al., 2019; Kumar et al., 2020; Tanaka et al., 2020; Zhang et al., 2024; Wang et al., 2026). Similar conclusions were obtained from the Shanlan rice landraces of Hainan, where population genomic analyses identified three distinct genetic groups and numerous novel genomic regions associated with cooking and eating quality, illustrating how geographically isolated farmer-managed populations continue to maintain unique genetic variation (Zhang et al., 2024). More recently, an analysis of 1,392 landraces collected from 18 Asian countries showed that traditional germplasm retained substantially greater nucleotide diversity than elite hybrid parental lines and contributed over 45% of favorable haplotypes identified for hybrid breeding, confirming that landraces remain an underexploited reservoir of useful variation for future crop improvement (Wang et al., 2026).

Evidence from regional conservation studies further demonstrates that traditional farming systems actively maintain rather than simply preserve diversity. In Japan, a core collection representing 87.5% of the diversity present in 236 traditional landraces retained sufficient variation for effective genome-wide association studies, illustrating that carefully selected landrace collections can capture extensive genetic diversity while remaining practical for breeding research (Tanaka et al., 2020). Studies of South Sudanese rice accessions likewise revealed low population differentiation (FST = 0.134) together with numerous previously undescribed grain-quality loci, emphasizing the contribution of locally adapted germplasm to broadening breeding populations (Mogga et al., 2018). In Yunnan Province, geographic and environmental gradients significantly influenced the genetic structure of japonica landraces, whereas environmental association analyses identified more than 1,300 loci associated with local adaptation, highlighting the importance of long-term farmer selection under contrasting ecological conditions (Hour et al., 2020; Cui et al., 2021). Comparable results were obtained in Guizhou, where approximately 73% of historical haplotypes were retained after 35 years of on-farm cultivation despite continuous selection by farmers, demonstrating that traditional management can conserve diversity while gradually enriching favorable alleles (Liu et al., 2022). Whole-genome comparisons of Yunnan landraces collected nearly three decades apart further showed minimal genome-wide divergence (FST = 0.026–0.036), indicating that on-farm conservation effectively maintains overall diversity while allowing adaptive selection to proceed (Cui et al., 2020). Thai purple rice landraces likewise exhibited strong population differentiation (overall FST = 0.841) corresponding closely to geographic origin, reflecting the maintenance of genetically distinct farmer-managed populations adapted to local environments (Pusadee et al., 2019). Collectively, these studies demonstrate that traditional cultivation systems are dynamic reservoirs of genetic diversity rather than static repositories, continuously generating valuable donor germplasm for climate-resilient, regionally adapted, and sustainable rice breeding.

### 6.3 Elite germplasm for low-GI and resistant-starch rice

Elite rice germplasm developed for reduced GI and elevated RS represents an important outcome of modern quality-oriented breeding. Unlike traditional landraces, these breeding materials have been developed through the integration of favorable genetic resources with desirable agronomic performance, resulting in improved nutritional quality while maintaining acceptable yield and grain characteristics (Cui et al., 2020; Liu et al., 2022). Recent breeding programs have identified elite lines that consistently exhibit increased resistant starch content, slower starch digestibility, and reduced predicted glycemic index compared with conventional cultivars, making them promising candidates for the development of functional rice products (Pusadee et al., 2019; Jing et al., 2023). In addition to improved nutritional properties, many elite lines retain acceptable cooking quality and processing characteristics, although considerable variation remains among breeding populations depending on the genetic background and breeding strategy employed (Selvaraj et al., 2021; Badoni et al., 2024). Consequently, elite low-GI germplasm provides an important bridge between the diverse genetic resources preserved in landraces and the development of commercially competitive rice cultivars with enhanced health benefits.

Compared with conventional cultivars, elite low-GI and high-RS germplasm consistently exhibits distinctive grain quality and nutritional characteristics. Numerous breeding studies have reported increased resistant starch content, reduced rapidly digestible starch, and lower predicted glycemic index in elite breeding lines, together with slower starch digestion rates in both *in vitro* and *in vivo* evaluations (Pusadee et al., 2019; Badoni et al., 2024). These nutritional improvements are frequently accompanied by firmer cooked texture, reduced stickiness, and improved starch stability during cooling, although the magnitude of these traits varies among breeding populations and genetic backgrounds (Hu et al., 2022; Liu et al., 2022). Considerable phenotypic diversity is also observed among elite lines with respect to cooking quality, processing performance, and grain appearance, indicating that multiple breeding strategies can achieve improved nutritional quality while maintaining acceptable consumer characteristics (Jing et al., 2023; Badoni et al., 2024).

Despite their nutritional advantages, elite high-RS germplasm often exhibits important agronomic and quality trade-offs. Many breeding populations show reductions in grain weight and yield potential together with increased chalkiness or changes in cooking quality compared with conventional cultivars (Pusadee et al., 2019; Hu et al., 2022; Liu et al., 2022). The magnitude of these trade-offs differs among breeding populations, indicating that favorable nutritional characteristics can be achieved without identical impacts on agronomic performance. Recent studies have therefore emphasized the importance of evaluating elite germplasm across diverse genetic backgrounds and production environments to identify lines combining improved nutritional quality with stable grain characteristics (Pusadee et al., 2019; Jing et al., 2023). From a nutritional perspective, rice with elevated resistant starch has been associated with slower glucose release, lower glycemic response, and increased production of beneficial short-chain fatty acids following colonic fermentation, supporting its potential value for developing functional food products (Wang et al., 2021; Badoni et al., 2024; Tiozon et al., 2024).

The performance of elite low-GI and high-resistant starch germplasm is influenced not only by its genetic composition but also by growing conditions and crop management. Evaluations conducted across multiple environments have shown that resistant starch content and grain quality may vary with temperature and grain-filling conditions, indicating that the expression of desirable nutritional traits is environment dependent (Itoh et al., 2003; Wang et al., 2021). Consequently, multi-location and multi-year field evaluations are essential for identifying elite germplasm that combines stable nutritional quality with consistent agronomic performance. Such validation is particularly important before incorporating promising lines into breeding programs or commercial cultivar development.

Although elite high-RS germplasm offers clear nutritional advantages, further improvement is still required before widespread commercial deployment. Many promising breeding lines continue to exhibit compromises in grain yield, seed weight, chalkiness, or consumer-preferred cooking quality compared with conventional cultivars (Selvaraj et al., 2021; Miura et al., 2022). These limitations highlight the need for continued evaluation of elite germplasm to identify donor lines that combine favorable nutritional traits with stable agronomic and market performance. Future breeding efforts should therefore focus on validating promising germplasm across diverse genetic backgrounds and production environments while integrating high nutritional quality with farmer and consumer requirements. Collectively, currently available elite germplasm provides an important foundation for developing the next generation of rice cultivars with improved health benefits, stable performance, and broad market acceptance.

## 7 Breeding strategies for amylose spectrum improvement

### 7.1 Marker-assisted selection

Marker-assisted selection (MAS) has long been considered one of the most efficient molecular breeding approaches because it enables indirect selection of favorable alleles through DNA markers, thereby increasing selection accuracy, shortening breeding cycles, and allowing genotype-based selection at the seedling stage with a theoretical heritability approaching 1.0. However, large-scale breeding experience has revealed that these theoretical advantages are not always translated into practical genetic gain—for example, an evaluation of more than 60 validated genes and QTLs in elite indica rice showed that nearly 50% were completely absent from breeding germplasm, 17% occurred at very low frequencies, and only **~**25% were readily available for routine forward breeding (Cobb et al., 2019). These findings indicate that the major limitation of MAS is no longer the identification of markers but their effective deployment into elite breeding populations. Nevertheless, recent studies demonstrate that integrating MAS with conventional breeding can substantially improve breeding efficiency—for instance, simultaneous pyramiding of four functional genes controlling aroma (BADEX7-5), panicle architecture (DEP1), grain length (FMGS-7), and grain width (GW8) produced a new basmati line with a higher grain yield (4.7 t ha^−1^), greater thousand-grain weight (28.5 g), and longer cooked grain length (19.5 mm) than the parental cultivars (Riaz et al., 2023). Collectively, these results suggest that the success of MAS depends less on marker availability than on robust marker validation, appropriate breeding strategies, and efficient gene pyramiding.

For many complex agronomic traits, the effectiveness of MAS is largely determined by the type and quality of molecular markers used for selection. Early breeding programs mainly relied on linked markers, which identify genomic regions associated with target loci but remain vulnerable to recombination, thereby reducing selection accuracy across different genetic backgrounds (Zhou et al., 2018; Arbelaez et al., 2019). Consequently, recent research has shifted toward functional markers developed directly from causal polymorphisms within target genes. Because these markers are in complete linkage with the functional allele, they provide more reliable genotype–phenotype prediction and substantially reduce false-positive selection (Zhou et al., 2018; Salgotra et al., 2020). The rapid development of SNP-based technologies has further accelerated this transition—for example, a core KASP platform achieved a 94.8% assay design success rate, generating 467 informative SNP markers from 596 candidate loci and successfully classifying 481 rice accessions into distinct genetic groups while simultaneously identifying markers associated with grain quality, yield, and resistance traits (TP et al., 2022). Similarly, functional marker development for the *Wx* locus enabled the rapid and inexpensive discrimination of alleles controlling intermediate amylose content, providing a practical tool for routine large-scale breeding populations (Salgotra et al., 2020). Collectively, these advances indicate that the current challenge in MAS is no longer marker discovery itself but the development of highly diagnostic, breeder-friendly marker systems that remain accurate across diverse germplasm and can be efficiently integrated into high-throughput breeding pipelines.

A major transition in modern MAS has been the integration of marker-assisted selection with complementary breeding strategies that increase both selection efficiency and breeding speed. Rather than using molecular markers as a stand-alone tool, current breeding programs combine MAS with rapid generation advance, speed breeding, high-throughput genotyping, and rigorous phenotypic evaluation. This integrated framework shortens breeding cycles while maintaining selection accuracy for complex traits (Cobb et al., 2019)—for example, SNP-assisted introgression of the *hst1* (*OsRR22*) salinity-tolerance allele combined with speed breeding produced a BC_3_F_3_ population within only 17 months across six generations. Whole-genome sequencing demonstrated 93.5% recovery of the recurrent parent genome, while the improved line exhibited significantly greater salt tolerance with minimal changes in agronomic performance under normal conditions (Rana et al., 2019). At the population level, integrating early-generation genotyping with phenotyping reduced the breeding costs by 25%–68% compared with conventional MAS while enabling the reliable identification of superior QTL combinations that consistently maintained a higher grain yield under drought across successive generations (Kumar et al., 2018b). The economic analyses further indicate that rapid generation advance consistently improves breeding efficiency and substantially increases the cost-effectiveness of MAS, particularly for traits controlled by major genes such as disease resistance and grain quality (Bonnecarrere et al., 2019). Nevertheless, successful implementation depends on careful integration rather than technology alone. Modern breeding frameworks emphasize validated QTLs, robust diagnostic markers, and efficient deployment into elite genetic backgrounds to minimize linkage drag and maximize the practical value of molecular selection (Cobb et al., 2019).

Overall, MAS has evolved from a simple marker-based screening approach into an integral component of modern molecular breeding. Its greatest value lies not only in identifying favorable alleles but also in enabling breeders to make accurate selection decisions before extensive field evaluation, thereby improving the efficiency of population advancement and reducing unnecessary phenotyping (Cobb et al., 2019). Recent developments have further expanded the scalability of MAS. A single seed-based marker-assisted selection strategy generated sufficient DNA (7 to 8 ng/μL) from individual rice seeds for at least 10 PCR marker assays, achieved approximately 99% genotypic concordance with conventional leaf sampling, increased SNP call rates (99.24% vs. 97.5%), and reduced sampling costs by approximately fourfold. These improvements allow desirable genotypes to be identified before planting while maintaining high genotyping accuracy (Arbelaez et al., 2019). Economic evaluations similarly indicate that integrating MAS with rapid generation advance provides greater breeding efficiency than conventional breeding alone, particularly for grain quality traits such as amylose content, where early molecular selection produces the greatest genetic gain per unit cost (Bonnecarrere et al., 2019). Therefore, MAS should be regarded as the foundation of modern molecular breeding, whereas its practical implementation for transferring favorable alleles into elite cultivars is achieved through marker-assisted backcrossing, which is discussed in the following section (Cobb et al., 2019).

### 7.2 Marker-assisted backcrossing

Whereas MAS provides a general framework for genotype-based selection, marker-assisted backcrossing (MABC) is a specialized breeding strategy designed to introgress favorable alleles into elite cultivars while rapidly recovering the recurrent parent genome and minimizing linkage drag (Liu et al., 2006; Xu, 2010). This strategy is particularly useful when a desirable-grain-quality allele is present in a donor line but must be introduced into an already adapted cultivar without substantially changing its overall agronomic performance.

The MABC process usually begins with a cross between an elite recurrent parent and a donor genotype carrying the target allele. The resulting F1 plants are backcrossed to the recurrent parent to generate BC1F1 progeny. At this stage, foreground selection is used to retain individuals carrying the target donor allele, whereas non-carrier plants are discarded before further backcrossing. Under Mendelian segregation, approximately half of the BC1F1 population is expected to retain the donor allele, making marker-based screening useful for reducing population size early in the breeding process (Iftekharuddaula et al., 2012; Cho et al., 2020). Selected BC1F1 plants are then backcrossed again to produce BC2F1 progeny. At this stage, foreground selection is combined with background selection to identify plants that both carry the target allele and contain a high proportion of recurrent parent genome. Without marker selection, BC2F1 plants are expected to carry approximately 87.5% recurrent genome, whereas marker-assisted background selection can increase this proportion to approximately 90%–95% by preferentially retaining plants with fewer donor segments outside the target region (Cho et al., 2020; Kim et al., 2021).

Further backcrossing produces BC3F1 populations in which recurrent parent genome recovery approaches near-isogenic levels. Although the theoretical recurrent genome recovery in BC3F1 populations is approximately 93.75%, marker-assisted background selection can increase this value to 96%–98% in selected individuals (Cho et al., 2020; Sreewongchai et al., 2024). Selected BC3F1 plants are then selfed to produce BC3F2 progenies, where the target allele segregates in a 1:2:1 ratio. Marker screening enables the identification of homozygous plants carrying the desired donor allele, which are then evaluated for amylose content, grain quality, and agronomic performance (Gao et al., 2009). Thus, MABC differs from general MAS by combining target allele retention with the systematic recovery of the elite genetic background.

Several breeding studies demonstrate the effectiveness of MABC for modifying amylose content while maintaining the agronomic performance of elite rice cultivars (**Table 4**). These studies show that MABC can reduce or increase amylose content while maintaining a high proportion of the recurrent parent genome—for example, improved restorer lines showed amylose reduction from 25.4% to 11.6%–23.5%, with recurrent genome recovery ranging from 81.1% to 92.6% (Riaz et al., 2023). Pseudo-backcross breeding for low-amylose and aroma traits achieved approximately 92% recurrent genome recovery in early generations (Cho et al., 2020), whereas high-amylose introgression into the CH1 background reached 97.76% recurrent genome recovery in selected BC1F2-derived material (Sreewongchai et al., 2024). Similarly, SNP-based MABC in japonica backgrounds produced selected BC2F1 lines with 97.4%–99.1% recurrent genome recovery and BC2F2 lines with amylose content of 12.4%–16.8% (Kim et al., 2021). Collectively, these examples show that MABC is most valuable when the breeding objective is not only to select a favorable allele but also to rebuild the elite cultivar background around that allele. Although MABC efficiently transfers individual favorable alleles into elite backgrounds, the simultaneous improvement of several agronomic and grain quality traits often requires the pyramiding of multiple genes, which is discussed in the following section.

**Table 4:** Representative MABC and pseudo-backcross breeding studies for modification of amylose content and grain quality in rice.

Study	Donor parent	Recurrent parent	Target allele/trait	Foreground marker(s)	Background selection	Generations	Recurrent genome recovery	Amylose outcome	Key significance
Cho et al., 2020 (Cho et al., 2020)	RNP20-145-1-9	Sinthukha	Low amylose and aroma	*Wx* and *badh2* markers	AFLP-based background selection	BC1F1 to BC1F2 (pseudo-backcross)	84% in BC1F1 and 92% in BC1F2	Selected plants showed low-amylose phenotype similar to donor, numeric AC NR	Showed that pseudo-backcross breeding can rapidly recover recurrent background while improving amylose and aroma
Sreewongchai et al., 2024 (Sreewongchai et al., 2024)	RD49	CH1	High amylose	OSR19 (*Wx*-specific)	67 SSR markers	BC1F1 to BC1F3 (pseudo-backcross)	91.04% in selected BC1F1 and 97.76% in selected BC1F2	High-amylose phenotype maintained (numeric AC not reported)	Demonstrated efficient introgression of high-amylose trait with rapid recurrent background recovery
Kim et al., 2021 (Kim et al., 2021)	Low-amylose donor lines	Samgwang and Milkyqueen	*SSIIIa* allele for starch quality improvement	Trait-specific SNP marker for *SSIIIa*	386 KASP markers	BC1F1, BC2F1, BC2F2	97.4%–99.1% in selected BC2F1 lines	BC2F2 lines showed AC of 12.4%–16.8%	Demonstrated high-efficiency MABC using dense SNP-based background selection for rapid recovery of elite background
Zhang et al., 2005 (Yang et al., 2019)	R367, 91499, Yanhui 559, Hui 527	57	Reduction of high AC	PCR-AccI marker for *Wx* genotype discrimination	NR	Backcross program reported, exact BC generation NR	NR	GG >20%, GT 17.71%–28.48%, TT <18%	Showed that *Wx* splice-site marker-assisted selection can lower AC, but phenotype remains influenced by genetic background

### 7.3 Gene pyramiding

Gene pyramiding, the simultaneous introgression of multiple favorable alleles into a single genetic background, represents an advanced MAS strategy for developing rice cultivars that combine desirable amylose levels with improved agronomic and nutritional traits (Xu, 2010). Because amylose content strongly influences both eating quality and metabolic properties, breeding programs increasingly aim to integrate favorable *Wx* alleles with genes controlling yield, stress tolerance, and grain quality (Yang et al., 2019). This strategy addresses a common challenge in rice improvement, namely, the need to balance nutritional enhancement with agronomic performance and consumer acceptance (Lou et al., 2026).

One major application of gene pyramiding involves combining favorable *Wx* alleles with yield-related genes such as *Gn1a*, *GS3*, and *DEP1*, which control grain number, grain size, and panicle architecture (Guillén et al., 2018; Adhikari and Poudel, 2020). Such combinations allow breeders to develop high-yielding cultivars while maintaining desirable amylose levels, thereby overcoming the frequent trade-off between grain quality and yield potential observed in some high-amylose genotypes (Zhu et al., 2012). Similar strategies have been applied to combine amylose-related loci with stress tolerance genes or QTLs associated with drought, salinity, or temperature stability of amylose content, including qHAC4, qHAC8a, and qHAC8b (Xu, 2010; Zhang et al., 2014)—for example, Xiao et al. demonstrated that blast resistance genes could be successfully integrated with low-amylose alleles using MAS, illustrating the feasibility of simultaneous improvement of grain quality and disease resistance (Xiao et al., 2018).

Gene pyramiding is also widely used to optimize cooking and eating quality traits by combining *Wx* alleles with additional grain quality loci. A particularly important interaction occurs between *Wx* and *ALK* (*SSIIa*), which controls starch gelatinization temperature and strongly influences cooking properties (Sharma and Khanna, 2019). Specific allele combinations of *Wx* and *ALK* enable breeders to fine-tune grain texture and cooking performance for different consumer markets. Studies have also identified favorable haplotype combinations of *Wx*, *ALK*, and other grain quality genes that produce rice varieties with moderate amylose content, desirable texture, and improved glycemic properties—for instance, combinations of intermediate amylose alleles (*Wx[b]* or *Wx[in]*) with suitable *ALK* variants and soft gel consistency traits have been proposed as optimal configurations for developing low-GI rice varieties acceptable to South Asian consumers (Selvaraj et al., 2021).

In practical breeding programs, gene pyramiding typically involves identifying target genes through QTL mapping or functional genomics, developing diagnostic markers for each locus, and designing crossing schemes that combine favorable alleles from multiple parental lines. Marker-assisted foreground selection is then applied to track all target genes simultaneously, while background selection minimizes linkage drag and accelerates the recovery of elite genetic background. Despite its effectiveness, pyramiding multiple genes presents several challenges, including the need to screen large populations to recover rare multi-gene combinations, the potential presence of unfavorable linkage blocks, and epistatic interactions that may reduce additive genetic effects (Xu, 2010). Nevertheless, gene pyramiding remains a powerful strategy for integrating grain quality improvement with yield, resilience, and nutritional traits in modern rice breeding programs (Riaz et al., 2023; Mynbayeva et al., 2024). Unlike MAS, MABC, and gene pyramiding, which rely on recombination and selection of naturally occurring alleles, precise breeding technologies enable the direct modification of target genes and are discussed in the following section.

### 7.4 Phenotyping

Efficient breeding for optimal amylose content requires phenotyping strategies that balance throughput, cost, and analytical precision across different breeding stages. Modern programs increasingly integrate molecular genotyping with tiered phenotyping approaches in which molecular markers guide early selection, while detailed phenotypic evaluation is reserved for advanced breeding lines (Lou et al., 2026). This combination reduces costs and accelerates breeding progress while maintaining reliable assessment of grain quality traits.

In early segregating generations such as F2, BC1F1, and BC2F1, phenotyping for amylose content is usually limited because seed availability is low and marker-based selection provides efficient identification of favorable *Wx* alleles (Liu et al., 2006). Genotyping using markers such as RM190, CAPS, or KASP therefore dominates selection at this stage. As breeding lines advance to intermediate generations including BC3F2 or F4–F5, rapid screening methods such as iodine staining become useful for verifying genotype-based predictions and identifying outlier lines (Xu, 2010; Zhang et al., 2018). The iodine colorimetric assay relies on the formation of a blue amylose–iodine complex and provides semi-quantitative estimates of amylose content from small grain samples within a few hours (Gauda et al., 2023). Visual iodine staining of endosperm sections can also differentiate waxy, low-, intermediate-, and high-amylose phenotypes (Hwang et al., 2005; Zhang et al., 2018).

Advanced breeding lines undergo more comprehensive phenotyping to evaluate starch functionality and consumer-relevant grain quality traits. Standardized amylose quantification, resistant starch analysis after cooking and cooling, Rapid Visco Analyzer (RVA) profiling of starch pasting properties, and near-infrared reflectance spectroscopy (NIRS) are commonly applied at this stage (Sun et al., 2017; Fu et al., 2022). Cooking protocols used for resistant starch analysis typically include controlled rice-to-water ratios followed by cooling at 4°C to promote starch retrogradation, which contributes to resistant starch formation and influences glycemic response (Guillén et al., 2018). RVA measurements provide detailed information on gelatinization and retrogradation behavior, with high-amylose rice generally showing higher setback viscosity and stronger retrogradation tendencies (Sun et al., 2017; Fu et al., 2022).

High-throughput phenotyping platforms are increasingly integrated with genomic selection (GS), which uses genome-wide marker information to predict breeding values for complex traits. In the case of amylose-related quality traits, GS models trained on phenotyped populations can predict amylose content with moderate to high accuracy, enabling early selection in breeding populations without extensive phenotyping. Combining MAS for major genes such as *Wx* and *ALK* with genomic selection for polygenic traits including yield and stress tolerance represents a promising strategy for accelerating the development of rice cultivars with optimized grain quality and nutritional properties (Shao et al., 2021).

## 8 Precision breeding

### 8.1 CRISPR editing of *SBEIIb*

CRISPR/Cas9-mediated genome editing of *SBEIIb* has emerged as a powerful strategy for generating rice varieties with substantially elevated amylose and resistant starch content. Unlike conventional breeding approaches that rely on naturally occurring allelic variation, genome editing enables targeted disruption of genes controlling starch branching, thereby producing predictable changes in starch structure and functionality (Sun et al., 2017; Li et al., 2021). Because *SBEIIb* plays a central role in amylopectin branching, loss-of-function mutations in this gene shift starch composition toward higher amylose and increased resistant starch formation.

Early studies demonstrated the feasibility of this approach through CRISPR/Cas9-mediated mutagenesis of *SBEI* and *SBEIIb*, which generated homozygous or biallelic mutant rice lines in the T0 generation. After segregation of the editing construct in the T1 generation, transgene-free plants were recovered, addressing regulatory concerns associated with transgenic crops (Sun et al., 2017). Phenotypic characterization of *SBEIIb* mutants revealed dramatic increases in amylose content reaching approximately 30%–40% and resistant starch levels of 10%–15%, accompanied by changes in pasting properties detected by Rapid Visco Analyzer profiling. Structural analyses further showed altered starch granule morphology with irregular shapes and surface fissures, reflecting reduced amylopectin branching and increased amylose accumulation. However, these nutritional improvements are accompanied by important agronomic trade-offs. *SBEIIb*-deficient lines frequently exhibit abnormal starch granule organization, reduced grain weight, and impaired grain filling due to disrupted starch biosynthesis. Consequently, although resistant starch content is substantially increased, grain appearance, processing quality, and overall yield may be negatively affected, highlighting the need to balance nutritional enhancement with acceptable agronomic performance (Sun et al., 2017; Gauda et al., 2023).

The nutritional implications of these edited lines are significant. Rice lines deficient in *SBEIIb*, particularly when combined with mutations in starch synthase IIIa, exhibit substantial increases in resistant starch content and reduced postprandial glycemic responses (Tanaka et al., 2018; Saito et al., 2020). Experimental studies in animal models and human subjects indicate that such high-resistant-starch rice can improve insulin regulation and mitigate hyperglycemic responses after consumption. Environmental factors may further influence these effects, as elevated temperatures during grain filling have been shown to enhance resistant starch formation in certain mutant backgrounds (Zhou et al., 2022). Therefore, future applications of CRISPR-mediated *SBEIIb* editing should aim to maximize nutritional benefits while minimizing undesirable effects on grain morphology, grain weight, and yield. These findings highlight the potential of genome editing to produce rice varieties with improved metabolic properties and targeted nutritional benefits.

### 8.2 Fine-tuning *Wx* expression

Beyond gene knockout strategies, CRISPR technology also enables precise modulation of gene expression through editing of cis-regulatory elements. Targeted modification of promoter regions and untranslated regulatory sequences of the *Wx* gene allows breeders to fine-tune amylose levels without completely abolishing gene function (Yang et al., 2022; Zhang et al., 2022). This regulatory editing approach provides a powerful means of generating a continuum of amylose phenotypes tailored to specific culinary or nutritional preferences.

Recent studies have demonstrated the effectiveness of this strategy by editing regulatory regions controlling *Wx* transcription. Genome editing of the promoter region was shown to modulate *Wx* expression levels and generate a spectrum of amylose contents ranging from approximately 5% to 20% (Zhang et al., 2022). Such variation is particularly valuable for developing soft-textured rice varieties with very low amylose levels that exhibit desirable eating quality in many consumer markets. Similarly, CRISPR/Cas9 editing of the intron 1 splice donor site (5′UISS) located within the 5′ untranslated region (5′UTR) of *Wx* has been used to reduce pre-mRNA splicing efficiency, thereby decreasing *Wx* expression and amylose accumulation in *indica* rice lines while maintaining overall agronomic performance (Yang et al., 2022).

More advanced regulatory editing approaches have generated novel *Wx* alleles with altered transcriptional patterns and temperature-responsive expression. Promoter editing experiments have produced multiple new allele variants that significantly modify amylose content and cooking properties (Zhao et al., 2024; Yan et al., 2025). Base editing technologies further enhance precision by introducing single nucleotide substitutions without generating double-strand breaks, thereby reducing off-target effects and improving editing efficiency (He et al., 2020). These innovations enable the rational design of rice varieties with optimized starch composition and stable grain quality across diverse environments.

### 8.3 Waxy rice development

Genome editing also provides a rapid method for developing glutinous rice varieties through targeted disruption of the *Wx* gene. Traditional breeding of waxy rice requires extensive backcrossing to transfer loss-of-function *wx* alleles into elite genetic backgrounds. In contrast, CRISPR/Cas9-mediated editing allows direct creation of *wx* mutants in high-performing cultivars. Targeted mutagenesis of the *Wx* gene has successfully generated waxy rice lines in several elite japonica varieties by introducing frameshift mutations in coding regions of the gene. These mutations drastically reduce amylose accumulation and convert conventional rice into glutinous rice without affecting key agronomic traits. Phenotypic evaluation of edited lines typically involves iodine staining of endosperm tissues, quantitative measurement of amylose content, and microscopic examination of starch granule morphology. Importantly, segregation of the CRISPR/Cas9 construct in subsequent generations allows the production of non-transgenic edited lines suitable for breeding applications (Zhang et al., 2018). Additional studies have confirmed the efficiency of this approach by generating null mutations in the *Wx* coding sequence that result in extremely low amylose levels of approximately 0%–2% and characteristic starch properties associated with glutinous rice (Fu et al., 2022). Genome editing has also been applied to create thermosensitive genic male sterile waxy rice lines, enabling the production of hybrid waxy rice varieties that combine improved yield potential with specialty starch characteristics (Zhu et al., 2025). Such developments demonstrate how precision genome editing can accelerate the creation of novel rice products that would be difficult to obtain through conventional breeding.

### 8.4 Regulatory and adoption landscape

Despite the rapid technological progress in genome editing, the regulatory landscape for CRISPR-edited crops remains heterogeneous across regions. In several countries, including the United States, Argentina, and Brazil, gene-edited plants that do not contain foreign DNA are not regulated as genetically modified organisms, which facilitates commercialization of edited crop varieties (Adhikari and Poudel, 2020; Li et al., 2021). In contrast, regulatory frameworks in the European Union and certain Asian countries treat genome-edited crops similarly to conventional transgenic organisms, creating additional regulatory hurdles for adoption (Li et al., 2021).

One important factor influencing regulatory acceptance is the ability to segregate the CRISPR/Cas9 construct from edited plants in subsequent generations. When the editing machinery is removed through Mendelian segregation, the resulting plants contain only small nucleotide changes indistinguishable from naturally occurring mutations (Sun et al., 2017; Zhang et al., 2018). This characteristic has strengthened arguments for regulatory exemption of many genome-edited crops and may accelerate the deployment of nutritionally enhanced rice varieties.

Public acceptance and intellectual property considerations also play important roles in the adoption of genome-edited crops. Consumer acceptance is likely to depend on clear communication of the benefits associated with precision breeding, particularly for health-oriented traits such as high-resistant-starch or low-glycemic-index rice (Adhikari and Poudel, 2020; Li et al., 2024). In addition, although CRISPR technology is protected by several patent frameworks, public-sector initiatives and humanitarian licensing programs have expanded access to genome editing tools for crop improvement in developing regions (Adhikari and Poudel, 2020; Li et al., 2021; Li et al., 2024). These factors will together influence how rapidly precision-bred rice varieties become integrated into global agricultural systems.

## 9 Integrated framework and future perspectives

The integration of genomics, physiology, and breeding provides a coherent framework for translating molecular knowledge into stable improvements in rice grain quality. This integration is most effective when gene-level information is linked to physiological processes and validated through breeding pipelines across environments. Genetic variation in key starch biosynthesis genes such as *Wx* and *SSIIa* establishes the foundation for amylose content and thermal properties. However, their phenotypic expression depends on regulatory networks, enzyme activity, and environmental conditions. Therefore, a systems-level perspective is required to ensure that molecular changes lead to predictable and stable grain quality outcomes (Bao, 2012; Fujita et al., 2022). A central principle of this integrated framework is the translation of allelic variation into measurable physiological traits. Variation in *Wx* directly affects GBSSI activity and amylose synthesis, while *SSIIa* influences gelatinization temperature through amylopectin structure. These genetic effects propagate through intermediate phenotypes such as starch composition, crystallinity, and granule morphology, and ultimately determine cooking and eating quality. This hierarchical relationship allows breeders to select at multiple levels, from molecular markers to functional assays such as AAC and RVA profiles. Importantly, polygenic interactions among *Wx*, *SSIIa*, and branching enzymes create emergent properties that cannot be predicted from single genes alone (Fujita et al., 2022).

To operationalize this knowledge, breeding strategies must combine marker-assisted selection with physiological validation. Functional markers targeting causal polymorphisms in *Wx* and *SSIIa* enable efficient early-generation selection. At the same time, haplotype-based approaches capture multi-locus effects and improve prediction accuracy across genetic backgrounds (Selvaraj et al., 2021). Gene pyramiding further enhances predictability by assembling favorable allele combinations across the starch biosynthesis pathway. However, molecular selection alone is insufficient. Physiological phenotyping remains essential to confirm that selected genotypes express the desired traits under realistic conditions (Xu et al., 2013).

Rather than functioning as independent technologies, modern molecular breeding approaches should be considered complementary components of an integrated framework for rice improvement. Marker-assisted selection is most effective for the rapid deployment of validated major-effect alleles, whereas marker-assisted backcrossing facilitates their transfer into elite genetic backgrounds while minimizing linkage drag. Gene pyramiding extends this strategy by combining favorable alleles controlling grain quality, yield, and stress tolerance within a single genotype. In parallel, GWAS, haplotype analysis, and systems genetics expand the pool of useful genetic variation by identifying superior alleles, regulatory networks, and environment-stable targets across diverse germplasm (Selvaraj et al., 2021). Multi-omics approaches further improve biological understanding by linking genetic variation with molecular and physiological mechanisms, thereby supporting more informed breeding decisions. Consequently, future breeding progress will depend not on the sequential application of individual methods but on their rational integration according to specific breeding objectives, trait architecture, and environmental conditions.

Despite these advances, several bottlenecks limit the translation of molecular data into breeding outcomes. First, starch biosynthesis is a complex network, and single-gene approaches often fail to capture this complexity. Second, genotype × environment interactions reduce the stability of many QTLs, particularly under heat stress during grain filling (Zhang et al., 2014). Third, small-effect loci and epistatic interactions complicate selection and require large populations or haplotype-based approaches. In addition, pleiotropy can introduce trade-offs between grain quality, yield, and stress tolerance. Finally, validation gaps persist between discovery in experimental populations and deployment in elite breeding materials (Zhao et al., 2024).

Multi-omics approaches provide powerful tools to address some of these challenges. Genomics identifies causal alleles and genetic architecture. Transcriptomics reveals regulatory responses and compensatory mechanisms. Metabolomics captures flux changes and pathway rerouting under stress. When integrated, these layers uncover regulatory nodes and epistatic interactions that shape amylose accumulation (Baysal et al., 2020; Ying et al., 2025). However, their practical application is limited by cost, data complexity, and environmental sensitivity. Therefore, multi-omics currently serves primarily for mechanism discovery and target identification rather than routine breeding.

Climate variability adds another layer of complexity to gene-to-phenotype translation. High temperature during grain filling disrupts *Wx* pre-mRNA splicing, reduces GBSSI levels, and decreases amylose content (Zhang et al., 2014). Combined heat and drought stress further exacerbate these effects by limiting substrate availability and enzyme activity. As a result, breeding for stable grain quality requires explicit consideration of environmental factors. Multi-environment testing is essential to identify genotypes with consistent performance. In this context, loci such as qHAC and qSAC3, which stabilize amylose under stress, represent valuable targets for breeding (Zhang et al., 2019).

Importantly, trade-offs between stress tolerance, yield, and grain quality are not inevitable. Evidence shows that careful locus selection and appropriate genetic backgrounds can minimize these trade-offs (Dixit et al., 2020; Yadav et al., 2024). Tissue-specific regulation provides an additional strategy to resolve conflicts. For example, endosperm-specific modulation of regulatory genes can stabilize amylose without affecting fertility (Cui et al., 2010). These approaches highlight the importance of precise genetic and physiological interventions.

Future rice breeding should move beyond improving individual traits toward the integrated design of grain quality ideotypes. Rather than optimizing amylose content alone, future breeding strategies should simultaneously balance starch composition, eating quality, resistant starch, yield stability, and climate resilience. Achieving this objective will require coordinated use of molecular breeding, genome editing, physiological phenotyping, and predictive breeding models that incorporate environmental information. High-throughput phenotyping, genomic selection, and machine learning will further improve the identification of superior genotypes by integrating molecular, physiological, and environmental data. In parallel, CRISPR-based editing provides opportunities to create or fine-tune alleles that are absent from natural germplasm, while multi-omics analyses help identify regulatory networks underlying complex quality traits. These complementary approaches together support an integrated breeding framework that enables the rational design of rice cultivars adapted to future production systems rather than the optimization of individual traits in isolation (Adhikari and Poudel, 2020; He et al., 2020; Li et al., 2021).

The integration of genomics, physiology, and breeding represents a mature but continuously evolving paradigm for rice quality improvement. Major genes such as *Wx* and *SSIIa* provide a strong foundation for predicting amylose-related traits, but stable and climate-resilient outcomes also require consideration of polygenic interactions, environmental effects, and physiological mechanisms. Future progress will depend less on the development of additional molecular tools than on their rational integration into breeding strategies that simultaneously optimize grain quality, agronomic performance, and environmental stability. Such an integrated framework provides a practical roadmap for developing rice cultivars that meet future demands for nutrition, consumer preference, and sustainable agricultural production.

## 10 Conclusions

AC should no longer be regarded as a trait determined primarily by the *Wx* gene but rather as a systems-level phenotype emerging from coordinated interactions among starch biosynthesis genes, physiological processes, and environmental conditions during grain development. Although *Wx* remains the principal determinant of GBSSI activity and amylose synthesis, increasing evidence demonstrates that enzymes such as *SBEIIb*, *SSIIa*, and *SSIIIa*, together with regulatory mechanisms controlling transcription, pre-mRNA splicing, and protein activity, collectively shape starch composition. These multilayered interactions explain the continuous spectrum of amylose phenotypes observed across rice germplasm and highlight the need to consider both molecular and physiological regulation when improving grain quality.

The extensive diversity present in rice germplasm, including landraces, elite breeding materials, and specialized high-resistant-starch lines, provides a valuable genetic foundation for developing cultivars with tailored starch composition. Exploiting this diversity through systematic characterization of functional alleles and favorable haplotypes will facilitate the development of rice varieties that satisfy diverse consumer preferences while maintaining agronomic performance and nutritional value.

Rice breeding has evolved from phenotype-based selection toward predictive molecular design of starch composition. Conventional molecular breeding approaches, including marker-assisted selection, marker-assisted backcrossing, and gene pyramiding, together with precision genome editing, should be regarded as complementary rather than competing strategies. Marker-assisted approaches efficiently deploy naturally occurring favorable alleles into elite backgrounds, whereas genome editing enables the creation or fine-tuning of alleles that are absent from natural germplasm. Integrating these technologies provides greater breeding efficiency than reliance on any individual approach alone.

However, successful optimization of amylose content requires more than genetic manipulation of starch biosynthesis genes. Strong genotype × environment interactions, particularly during grain filling, substantially influence *Wx* expression, starch biosynthesis, and final grain quality. Future breeding programs should therefore integrate haplotype-based selection for major starch biosynthesis genes with genomic selection models trained using multi-environment phenotypic datasets. Such models can simultaneously predict amylose content, grain quality, yield stability, and environmental adaptation before extensive field evaluation, thereby reducing breeding population size, shortening selection cycles, and lowering phenotyping costs. Multi-omics approaches should primarily serve as discovery platforms for identifying causal regulatory networks and candidate genes, whereas routine breeding can rely on low-cost diagnostic molecular markers developed from these discoveries.
